# Evidence for genetic heterogeneity between clinical subtypes of bipolar disorder

**DOI:** 10.1038/tp.2016.242

**Published:** 2017-01-10

**Authors:** A W Charney, D M Ruderfer, E A Stahl, J L Moran, K Chambert, R A Belliveau, L Forty, K Gordon-Smith, A Di Florio, P H Lee, E J Bromet, P F Buckley, M A Escamilla, A H Fanous, L J Fochtmann, D S Lehrer, D Malaspina, S R Marder, C P Morley, H Nicolini, D O Perkins, J J Rakofsky, M H Rapaport, H Medeiros, J L Sobell, E K Green, L Backlund, S E Bergen, A Juréus, M Schalling, P Lichtenstein, P Roussos, J A Knowles, I Jones, L A Jones, C M Hultman, R H Perlis, S M Purcell, S A McCarroll, C N Pato, M T Pato, N Craddock, M Landén, J W Smoller, P Sklar

**Affiliations:** 1 Department of Psychiatry, Icahn School of Medicine at Mount Sinai, One Gustave L. Levy Place, New York, NY, USA; 2 Institute for Genomics and Multiscale Biology, Department of Genetics and Genomic Sciences, Icahn School of Medicine at Mount Sinai, One Gustave L. Levy Place, New York, NY, USA; 3 Stanley Center for Psychiatric Research, Broad Institute of Harvard and MIT, Cambridge, MA, USA; 4 MRC Centre for Psychiatric Genetics and Genomics, Cardiff Unviersity, Cardiff, UK; 5 Department of Psychological Medicine, University of Worcester, Worcester, UK; 6 Department of Psychiatry, University of North Carolina at Chapel Hill, Chapel Hill, NC, USA; 7 Department of Psychiatry, Harvard Medical School, Boston, MA, USA; 8 Center for Human Genetic Research, Massachusetts General Hospital, Boston, MA, USA; 9 Department of Psychiatry, Stony Brook University, Stony Brook, NY, USA; 10 Department of Psychiatry, Georgia Regents University Medical Center, Augusta, GA, USA; 11 Center of Excellence in Neuroscience, Department of Psychiatry, Texas Tech University Health Sciences Center at El Paso, El Paso, TX, USA; 12 Department of Psychiatry, Veterans Administration Medical Center, Washington, DC, USA; 13 Department of Psychiatry, Georgetown University, Washington, DC, USA; 14 Department of Psychiatry, Wright State University, Dayton, OH, USA; 15 Department of Psychiatry, New York University, New York, NY, USA; 16 Department of Psychiatry, University of California, Los Angeles, Los Angeles, CA, USA; 17 Department of Psychiatry and Behavioral Science, State University of New York, Upstate Medical University, Syracuse, NY, USA; 18 Departments of Family Medicine, State University of New York, Upstate Medical University, Syracuse, NY, USA; 19 Department of Public Health and Preventive Medicine, State University of New York, Upstate Medical University, Syracuse, NY, USA; 20 Center for Genomic Sciences, Universidad Autónoma de la Ciudad de México, Mexico City, Mexico; 21 Department of Psychiatry, Carracci Medical Group, Mexico City, Mexico; 22 Department of Psychiatry and Behavioral Sciences, Emory University, Atlanta, GA, USA; 23 Department of Psychiatry and the Behavioral Sciences, University of Southern California, Keck School of Medicine, Los Angeles, CA, USA; 24 School of Biomedical and Health Sciences, Plymouth University Peninsula Schools of Medicine and Dentistry, Plymouth University, Plymouth, UK; 25 Department of Clinical Neuroscience, Karolinska Institutet, Stockholm, Sweden; 26 Department of Molecular Medicine and Surgery, Karolinska Institutet, Stockholm, Sweden; 27 Department of Medical Epidemiology and Biostatistics, Karolinska Institutet, Stockholm, Sweden; 28 Friedman Brain Institute, Department of Neuroscience, Icahn School of Medicine at Mount Sinai, One Gustave L. Levy Place, New York, NY, USA; 29 Zilkha Neurogenetic Institute, University of Southern California, Keck School of Medicine, Los Angeles, CA, USA; 30 Center for Experimental Therapeutics, Massachusetts General Hospital, Boston, MA, USA; 31 Department of Genetics, Harvard Medical School, Boston, MA, USA; 32 Institute of Neuroscience and Physiology, Sahlgenska Academy at the Gothenburg University, Gothenburg, Sweden; 33 Psychiatric and Neurodevelopmental Genetics Unit, Center for Human Genetic Research, Massachusetts General Hospital, Boston, MA, USA

## Abstract

We performed a genome-wide association study of 6447 bipolar disorder (BD) cases and 12 639 controls from the International Cohort Collection for Bipolar Disorder (ICCBD). Meta-analysis was performed with prior results from the Psychiatric Genomics Consortium Bipolar Disorder Working Group for a combined sample of 13 902 cases and 19 279 controls. We identified eight genome-wide significant, associated regions, including a novel associated region on chromosome 10 (rs10884920; *P*=3.28 × 10^−8^) that includes the brain-enriched cytoskeleton protein adducin 3 (*ADD3),* a non-coding RNA, and a neuropeptide-specific aminopeptidase P (*XPNPEP1)*. Our large sample size allowed us to test the heritability and genetic correlation of BD subtypes and investigate their genetic overlap with schizophrenia and major depressive disorder. We found a significant difference in heritability of the two most common forms of BD (BD I SNP-*h*^2^=0.35; BD II SNP-*h*^2^=0.25; *P*=0.02). The genetic correlation between BD I and BD II was 0.78, whereas the genetic correlation was 0.97 when BD cohorts containing both types were compared. In addition, we demonstrated a significantly greater load of polygenic risk alleles for schizophrenia and BD in patients with BD I compared with patients with BD II, and a greater load of schizophrenia risk alleles in patients with the bipolar type of schizoaffective disorder compared with patients with either BD I or BD II. These results point to a partial difference in the genetic architecture of BD subtypes as currently defined.

## Introduction

Bipolar disorder (BD) is a mental illness characterized by episodes of mania and depression. Over the past century, the diagnostic criteria for this condition have evolved. First, ‘manic-depressive insanity’ was split from the condition that is today known as schizophrenia (SCZ).^1^ It was then split from what we now label major depressive disorder (MDD), and renamed BD.^2^ Subsequently, BD was formally divided into two clinical subtypes: bipolar I disorder (BD I), characterized by manic episodes; and bipolar II disorder (BD II), characterized by hypomanic episodes and recurrent depressive episodes.^3^ The initial BD I and BD II distinction was based primarily on a different longitudinal course, as differences in family loading^3^ and lithium responsiveness^4^ were not observed. The exact prevalence of each clinical subtype remains uncertain, with the most recent large epidemiological study reporting lifetime prevalence of 0.6% for BD I and 0.4% for BD II,^5^ similar to a recent systematic review^6^ but lower than previously reported.^7, 8^

Recurrence risk ratios for BD of 7–10 for first-degree relatives are observed, and several family studies have aimed to evaluate whether there is shared etiology for BD I and BD II. Some studies have shown familial co-aggregation of BD and MDD,^9^ but investigation of the specificity of familial aggregation of BD subtypes and their relationship to MDD to determine whether BD subtypes share etiology have yielded inconclusive results.^10, 11^ More recently, contemporary family studies have found familial aggregation of mania and major depressive episodes but not hypomania,^12^ and, similarly, of BD I and MDD but not BD II.^13^

In addition to BD I, a manic episode is required for the DSM-V diagnosis of schizoaffective disorder bipolar type (SAB). Since its initial description in 1933^14^ there has been debate regarding whether schizoaffective disorder (SA) is a form of SCZ, affective disorder, a combination of the two, or a separate entity altogether. BD and SCZ have historically been regarded as genetically distinct;^12, 15^ however, more recent studies have shown a significant shared genetic component of these disorders. A large population-based study of >35 000 patients with SCZ, 40 000 patients with BD and their family members found an increased risk of BD in first-degree relatives of SCZ probands, and vice versa.^16, 17^ Compared with SCZ and BD, there is evidence that SA shows weaker disorder-specific familial aggregation, with relatives of SA probands having relatively equivalent risk for SA, SCZ and BD.^15, 18^

Genetic studies of common variation in BD have identified multiple genome-wide significant associations between disease status and single nucleotide polymorphisms (SNPs).^19, 20, 21, 22, 23, 24, 25, 26, 27^ In 2011, the Psychiatric Genomics Consortium Bipolar Disorder Working Group (PGCBD) reported four loci meeting genome-wide significance, including regions in close proximity to genes *ODZ4, ANK3* and *SYNE1.*^24^ Subsequently two studies performed meta-analyses incorporating the PGCBD data and their own new samples: Chen *et al.*^26^ identified novel associations near the genes *TRANK1 (LBA1), LMAN2L* and *PTGFR*; Muhleisen *et al.*^27^ identified two new risk loci near *ADCY2* and a region between *MIR2113* and *POU3F2*. As demonstrated in other disorders, in particular SCZ,^28^ increasing sample size led to the identification of additional associated loci. Although each genome-wide association study (GWAS) only found a handful of genome-wide significant associations, it has been convincingly demonstrated that BD is polygenic; there are many common DNA variants whose effects are too small to detect individually, but when summed together, are contributing to BD risk.

Traditional family studies focus on relatives to determine the proportion of variance of liability to disease that is attributable to inherited genetic factors. In the absence of molecular data, these studies provide no information about the number, frequency or effect sizes of any genes or associated variants involved. With the emergence of genome-wide data for multiple psychiatric disorders, several methods have been developed for comparing conditions to one another at the genetic level. Three commonly used analytic approaches are comparative GWAS, polygenic scoring^29^ and SNP-based heritability estimation.^30, 31, 32^ Cross-disorder analysis of individual SNPs in SCZ and mood disorders (BD I, BD II, MDD) reported a genome-wide significant locus on chromosome 11 that appeared to be specific to BD II.^33^ Because there are many small-effect DNA variants, polygenic scoring methods were developed that derive a disease risk score for each individual in a data set by counting the number of previously identified risk alleles present. Polygenic scores derived from both SCZ- and MDD-risk alleles have discriminant ability between BD cases and controls.^17, 34^ SNP-based heritability methods, as opposed to utilizing genetic data only from loci previously implicated in disease, make genetic relatedness calculations by comparing all possible pairs of individuals in a data set at all genetic markers. When this information has been determined for two independent case–control data sets, the proportion of variance in phenotype explained by SNPs (labeled the SNP-based heritability, or SNP-*h*^2^) for each case trait and the genetic correlation (*r*_g_)^[ref. 35]^ between the case traits can be determined. Using this approach, SNP-*h*^2^ has been estimated to range between 0.20 and 0.25 for BD, SCZ and MDD, whereas the BD-MDD and BD-SCZ genetic correlations have been estimated at 50% and 70%, respectively.^30^ The effect of BD subtype composition on these estimates has not been investigated.

In order to markedly increase the available sample size for a GWAS and improve our power to identify risk loci and discern genetic differences across BD subtypes, we established the International Cohort Collection for Bipolar Disorder (ICCBD) with investigators from the United States (US), United Kingdom (UK) and Sweden. We performed a GWAS on the ICCBD sample, as well as a meta-analysis with the PGCBD for a total of 13 902 cases and 19 279 controls. Further, we took advantage of the size and composition of our sample to explicitly survey the genetic relationship across the clinical subtypes of BD (BD I, BD II and SAB). Specifically, we compare SNP-based measures of heritability and genetic correlation for BD I and BD II, and assess the polygenic loading of BD subtypes for risk alleles identified previously through large-scale GWAS of three psychiatric disorders (BD, SCZ and MDD).

## Materials and methods

### Subject ascertainment and sample collection

All procedures were approved by ethical committees at the Karolinska Institutet, University of Southern California and Cardiff University. All subjects provided written informed consent (or legal guardian consent and subject assent).

The ICCBD includes BD cases and unaffected controls from the Sweden Bipolar Disorder Cohort (SWEBIC), the Bipolar Disorder Research Network (BDRN) in the United Kingdom, and the Genomic Psychiatry Consortium (GPC) from the University of Southern California. Inter-rater reliability across sites was performed and showed agreement between trained clinicians on case status (Fleiss’ Kappa statistic for multiple raters *κ*=0.72 for distinguishing BD from other disorders based on case notes; see Supplementary Note). GWAS results have not been reported on 85.7% of the ICCBD case subjects. All of the SWEBIC and BDRN control subjects have been reported in prior GWAS of BD, schizophrenia and other disorders^36, 37^

SWEBIC controls are from the Swedish Schizophrenia Consortium.^36^ SWEBIC cases were identified through four channels: two national registries and two catchment areas. The Swedish National Quality Assurance Registry for Bipolar Disorder (BipoläR) led to the ascertainment of 1304 BD cases and the Swedish Hospital Discharge Register (HDR) yielded 233 BD cases. The HDR case subjects have been previously reported.^38^ Additional cases were obtained via two catchment areas: 271 cases were recruited via physician’s referral from the Affective Center at St. Göran Hospital in Stockholm and 493 cases were recruited from the greater Stockholm County region. SWEBIC controls were collected via the HDR and have been reported elsewhere.^36^ All BD diagnoses for the SWEBIC sample were made according to the DSM-IV criteria (Supplementary Note). Genotype analyses have been previously reported for a portion of the SWEBIC cases.^38^

Sample ascertainment strategies for the BDRN cases and controls have been previously reported^37, 39^ (Supplementary Note). BDRN controls are from the Wellcome Trust Case Control Consortium 2 (WTCCC2) control cohort.^37^ Case participants were interviewed using the Schedules for Clinical Assessment in Neuropsychiatry (SCAN).^40^ Psychiatric and general practice case-notes, where available, were also reviewed. On the basis of these data best-estimate lifetime diagnoses were made according to DSM-IV criteria and key clinical variables, such as age at onset and number of episodes, were rated. In cases where there was doubt, diagnostic and clinical ratings were made by at least two members of the research team blind to each other’s rating. Team members involved in the interview, rating and diagnostic procedures were all research psychologists or psychiatrists. Green *et al.*^39^ reported 1218 BDRN cases for 3106 SNPs with immunological annotations.

GPC cases and controls were collected via the University of Southern California healthcare system, as previously described.^41^ Using a combination of focused, direct interviews and data extraction from medical records, diagnoses were established using the OPCRIT.^42^ Age and gender-matched controls were ascertained from the University of Southern California health system and assessed using a validated screening instrument and medical records.

### Genotyping

For all ICCBD sites, DNA was extracted from peripheral blood samples that had been collected and stored at −20 °C. Samples were then genotyped at the Broad Institute. Genotypes were called using Birdsuite (Affymetrix, Santa Clara, CA, USA) or BeadStudio (Illumina, San Diego, CA, USA). Genotypes were generated as sufficient numbers of samples accumulated from field work (Supplementary Note).

### Quality control

**Figure 1:**
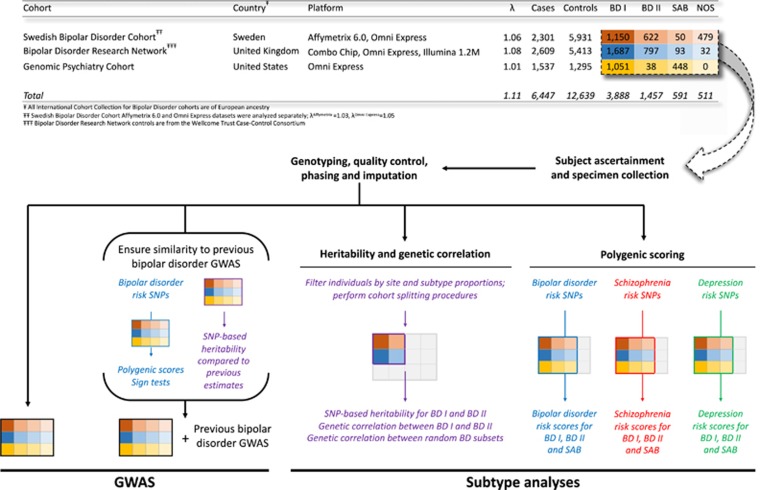
ICCBD sample description and analysis pipeline. The top panel (table) shows the composition of the ICCBD data set by site, genotyping platform and phenotype. The three ICCBD sites are represented by color, and the BD subtypes by shade. The bottom panel uses this representation of the dataset to diagram the workflow of the study and the portions of the ICCBD data set included in each analysis presented in the manuscript. Thickened borders designate the included subset, while excluded subsets are colored in grey. The coloring of text in the bottom panel is intended to illustrate how the same method (e.g., SNP-based heritability) was utilized for multiple purposes (i.e., ensuring similarity to previous GWAS, and comparing BD I to BD II). BD, bipolar disorder; GWAS, genome-wide association studies; ICCBD, International Cohort Collection for Bipolar Disorder; SNP, single-nucleotide polymorphism.

Data were processed by a quality control (QC) pipeline modeled after the central analysis pipeline of the PGCBD study.^24^ For each site, the goal was to create a set of genotyped SNPs of high and uniform quality maximizing the number of individuals retained. We first harmonized the SNP names, position and strand, then removed duplicated SNPs and individuals. SNPs with data missing in >5% of the sample were removed. Next, individuals with heterozygosity rate >15%, missingness rate >2%, or whose genotype-determined gender was ambiguous (0.25<F<0.75) were removed. SNPs on sex chromosomes were removed from analysis, and those remaining SNPs with data missing in >2% of the sample, minor allele frequency (MAF)<1%, or deviation from Hardy–Weinberg equilibrium (*P*<5 × 10^−5^) were removed. For data sets containing both case and control individuals, SNPs were also filtered based on differential missingness in cases compared with controls (*P*<1 × 10^−3^) or differential missingness based on haplotype (*P*<1 × 10^−10^). After these initial QC checks, SNPs were pruned based on linkage disequilibrium (window size=100, window shift=50 SNPs and VIF threshold=2), and multidimensional scaling (MDS) analysis on the N × N matrix of genome-wide identity-by-state pairwise distances was performed. Outlier individuals were identified using the ten most significant MDS components and excluded from the analysis so that cases and controls were appropriately matched. Relatedness between individuals in the population (defined as PIHAT value>0.1) was also evaluated, with one of the members being removed from the analysis when such relationships were identified. After all QC, there remained 6447 cases (1378 SWEBIC Illumina, 923 SWEBIC Affymetrix, 2609 BDRN, 1537 GPC) and 12 639 controls (3716 SWEBIC Illumina, 2215 SWEBIC Affymetrix, 5413 BDRN, 1295 GPC; Figure 1 and Supplementary Note).

### Phasing and imputation

After QC was completed on each of the ICCBD cohorts, phasing and imputation was performed. The data were phased using SHAPEIT^43^ and imputed using IMPUTE2,^44^ with phased 1000 Genomes world panel as reference. Each data set was imputed separately, splitting the data sets into 5MB imputation chunks. Following imputation, SNPs were filtered for MAF>0.01 and imputation quality score>0.3. After applying these filters to each cohort, meta-analysis was performed and only those SNPs with high-quality data in all cohorts were retained for analysis. This resulted in 8 886 502 SNPs for the ICCBD GWAS and 8 837 380 SNPs for the ICCBD–PGCBD meta-analysis.

### Sign test

We performed sign tests on a modified ICCBD sample where duplicates or related individuals between ICCBD and PGCBD had been removed (26 BDRN cases and 2610 BDRN controls). Sign tests were performed using index SNPs from the PGCBD data in approximate linkage equilibrium with association *P*-values below four thresholds (0.001, 1 × 10^−4^, 1 × 10^−5^ and 1 × 10^−6^).

### Polygenic scoring

We carried out polygenic scoring analyses^29^ on the ICCBD data set using the PGCBD discovery data set. Quantitative scores were computed for each ICCBD subject based on the set of SNPs with *P*-values less than predefined *P*-value thresholds (pT) in the discovery data set. For each SNP set defined by pT, we calculated the proportion of variance explained (Nagelkerke's *R*^2^) by subtracting the Nagelkerke's *R*^2^ attributable to ancestry covariates alone from the Nagelkerke's *R*^2^ for polygenic scores plus covariates.

We additionally performed polygenic scoring analyses for three different target BD subtypes within the ICCBD cohort: BD I; BD II; and SAB (Figures 1 and Supplementary Table 1). Polygene scores were calculated using three discovery datasets: non-overlapping sets derived from GWAS of SCZ (9087 cases, 12 171 controls; cases included a small fraction of SAB),^45^ BD (6704 cases, 9381 controls; cases were 85% BD I, 11% BD II and 4% SAB)^24^ and MDD (9041 cases, 9381 controls).^46^ The same set of controls was used in each of the target sets, and targets sets were filtered such that there were no overlaps with any of the discovery data sets. This yielded 3323 BD I cases, 1340 BD II cases, 570 SAB cases and 7814 controls for analysis. Quantitative scores were computed for each ICCBD subject at the defined pT and for each SNP set we calculated the proportion of variance explained (*R*^2^) by subtracting the Nagelkerke's *R^2^* attributable to ancestry covariates alone from the *R*^2^ for polygenic scores plus covariates. Next, using the same three discovery data sets, we performed the same polygene scoring procedure on target sets that, rather than containing ICCBD cases and controls, contained two ICCBD subtype cohorts. Three comparisons were performed in this manner: BD I vs BD II (3323 BD I cases, 1340 BD II cases), BD I vs SAB (3323 BD I cases, 570 SAB cases), and BD II vs SAB (1340 BD II cases, 570 SAB cases).

### Association analyses

All association analyses were conducted using logistic regression in PLINK.^32^ MDS was performed on the entire data set, and each collection wave was analyzed separately using as covariates those MDS components in the top 10 that were significantly correlated with phenotype. Results were then combined by meta-analysis in PLINK,^32^ and the heterogeneity *P*-values reported are those for the Cochrane’s Q statistic. Association analyses for BD were conducted using the 1000 Genomes imputed data. We used a genome-wide significance threshold of *P*<5 × 10^−8^. Prior to performing the ICCBD meta-analysis with the PGCBD, we reanalyzed the PGCBD data set (7481 cases and 9250 controls)^24^ using 1000 Genomes Project imputation, identifying no new genome-wide significant hits.

### Heritability analyses

Using methods previously described,^30, 31, 32^ we estimated the variance in liability explained by SNPs (SNP-*h*^2^) in the full ICCBD cohort and each individual site for BD and its subtypes (Figure 1 and Supplementary Tables 1–3). From the set of 8 886 502 SNPs included in the ICCBD GWAS, we further filtered to retain only SNPs that had imputation *R*^2^ of >0.8 and MAF>0.01 in all ICCBD samples, resulting in 7 252 417 SNPs upon which to calculate genome-wide similarity relationships between all pairs of individuals. For all SNP-*h*^2^ estimates reported, individuals were excluded to ensure that no pairs of individuals had a genome-wide similarity relationship >0.05. This procedure removes ancestry outliers in addition to those already removed in the standard GWAS quality control pipeline.

To fully dissect whether previous observations of SNP-*h*^2^ heterogeneity in BD^30^ were driven by subtype or site, further filtering was required to ensure that the composition of the case cohorts being compared were balanced in terms of these variables. Any subtype for which >75% of cases derived from a single ICCBD site were thus excluded from these analyses, limiting SNP-*h*^2^ subtype comparisons to BD I and BD II as 80% of SAB cases were from GPC (Figure 1 and Supplementary Table 1). Next, any site for which the relative contribution to the BD I and BD II case cohorts was grossly imbalanced was excluded. This removed GPC from the SNP-*h*^2^ subtype analyses (Figure 1 and Supplementary Table 1).

Having applied these site/subtype filters, BD I and BD II SNP-*h*^2^ were compared in the SWEBIC-BDRN cases using the full SWEBIC-BDRN control set to calculate each subtype SNP-*h*^2^ (Supplementary Table 3). We then applied a bivariate extension of these methods^35^ in order to estimate the genetic correlation (*r*_g_) explained by SNPs between BD I and BD II cases (*r*_g__-I/II_), and to test whether it differed from *r*_g_ between cohorts containing a mix of BD I and BD II cases (*r*_g__-mix_).

To avoid inflation of *r*_g_ estimates, the SWEBIC-BDRN controls used in the SNP-*h*^2^ calculations were randomly split into two evenly sized non-overlapping groups, one each for pairing with the case subsets being compared. We calculated 100 estimates of *r*_g__-I/II_, each using the same BD I (*n*=2811) and BD II (*n*=1,398) case groups but a unique permutation of the control splitting procedure. To calculate *r*_g__-mix_, for each of the 100 control group permutations used in calculating *r*_g__-I/II_ we combined the BD I and BD II cases into a single pool (*n*=4,209 cases) that was then randomly divided into two non-overlapping mixed case groups (that is, cohorts containing both BD I and BD II cases) sized according to the BD I and BD II case groups used in the *r*_g__-I/II_ estimates. We performed this case splitting procedure 100 times for each of the 100 control group permutations, for a total of 10 000 calculations of *r*_g__-mix_. To determine whether the distributions of *r*_g__-I/II_ and *r*_g__-mix_ estimates differed from one another, a *t*-test was performed.

## Results

### ICCBD GWAS and comparability to previous BD samples

Genome-wide SNP data of 6447 BD cases and 12 639 controls matched for ancestry were analyzed from a previously unreported population-based sampling in Sweden, the United Kingdom and the United States (Supplementary Figure 1). We performed logistic regression of case status on imputed dosages of 8 886 502 autosomal SNPs including sample and ancestry as covariates (see Materials and Methods). The resulting genomic inflation factor (*λ*)^47^ was 1.11 (Figure 1 and Supplementary Figure 2). Two regions met a genome-wide significance threshold of *P*<5 × 10^−8^: a 2 base-pair deletion on chromosome 9 (position 129209201, risk allele=TC, *P*=2.48 × 10^−8^, odds ratio (OR)=1.14), and a locus on chromosome 10 (position 111774807, rs10884920, risk allele=A, *P*=1.20 × 10^−8^, OR= 1.17; Supplementary Table 4 and Supplementary Figure 3). Neither variant has been previously reported to have an association with BD, MDD, or SCZ. Comparability of this new cohort was assessed in three ways. First, sign tests found that most ICCBD SNPs had the same direction of effect as the index PGCBD SNP at *P*<0.001 (66.73% in same direction, *P*=1.86 × 10^−28^; Supplementary Figure 4). Second, polygenic risk scoring found higher BD risk scores in cases in the full ICCBD sample (*P*=5 × 10^−89^; Supplementary Figure 5). Third, we estimated the BD SNP-based heritability (SNP-*h*^2^) using genome-wide complex trait analysis (GCTA)^48^ and found a SNP-*h*^2^ of 0.24 (s.e.=0.01; Supplementary Table 2), consistent with previous estimates from the PGCBD sample.^30^

### ICCBD–PGCBD meta-analysis

**Figure 2:**
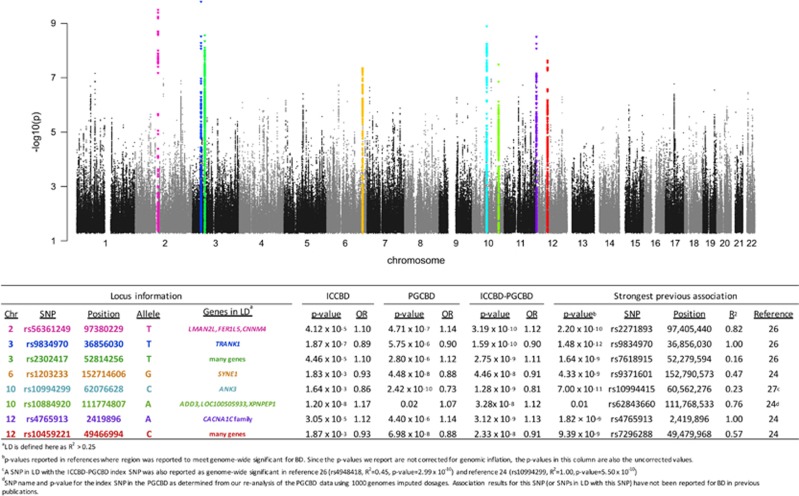
Association results for the ICCBD–PGCBD meta-analysis (13 902 cases, 19 279 controls). Horizontal axis shows chromosome and position. Vertical axis shows the –log10 *P*-value for association with BD. The table shows detailed statistics of the index SNP from each independent locus. The color of chromosome and SNP in the table corresponds the color of points for that locus in the Manhattan plot. BD, bipolar disorder; ICCBD, International Cohort Collection for Bipolar Disorder; PGCBD, the Psychiatric Genomics Consortium Bipolar Disorder Group; SNP, single-nucleotide polymorphism.

We combined the ICCBD cohort with PGCBD for a meta-analysis of 13 902 BD cases and 19 279 controls. Applying the same filters used in the ICCBD GWAS yielded 8 032 748 SNPs for analysis. There were 117 SNPs in 8 genomic regions that surpassed genome-wide significance (Figure 2 and Supplementary Figure 3). Of these, one region is novel; the chromosome 10 locus identified in the ICCBD GWAS (chromosome 10, position 111774807, rs10884920, *P*=3.28 × 10^−8^, heterogeneity *P*=0.27, OR=1.12). Among the genes in strong linkage disequilibrium with this locus are adducin3 (*ADD3)*, which codes for a ubiquitously expressed cytoskeletal protein implicated in cerebral palsy,^49^ and aminopeptidase P (*XPNPEP1)*, which has been found to be involved in the degradation and maturation of neuropeptides.^50^ Of the seven other genome-wide significant signals, four were reported in the PGCBD^24^ and an additional three by Chen *et al.*^26^ (Figure 2 and Supplementary Table 5). When these eight loci were tested for association with BD subtypes in the ICCBD cohort, the threshold for nominal significance (*P*<0.05) was surpassed for all loci in BD I, four loci in BD II and two loci in SAB (Supplementary Table 6). Moreover, the direction of effect was the same as in the ICCBD–PGCBD for all eight loci with respect to BD I and BD II, and for seven of the eight loci with respect to SAB (Supplementary Table 6).

### Heritability of BD I and BD II

**Figure 3:**
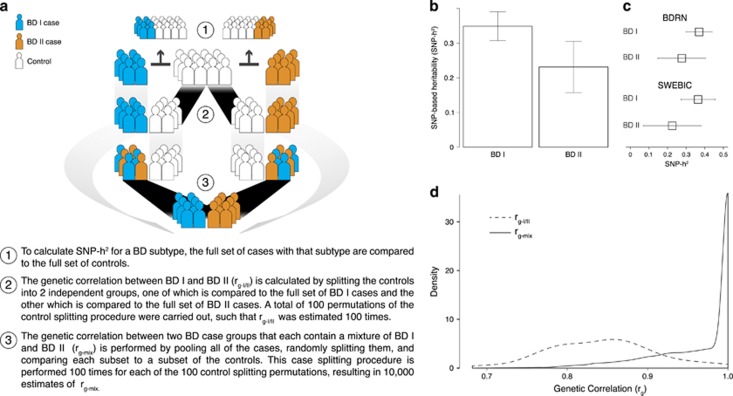
SNP heritability and genetic correlation estimations for BD I and BD II. We estimated the SNP-h^2^ for BD I (*n*=2,811 cases) and BD II (*n*=1398 cases), as well as the genetic correlation (r_g_) explained by SNPs between BD I and BD II (r_g-I/II_) and between random subsets of BD cases (r_g-mix_). (**a**) Sample splitting procedures for SNP-based heritability and genetic correlation estimation. (**b**) SNP-based heritability estimates for BD I and BD II. We observed a significant difference in the SNP-h^2^ of BD I and BD II (BD I SNP-h^2^=0.35, SE=0.02; BD II SNP-h^2^=0.25, SE=0.04; two-sided *t*-test *P*=0.02). (**c**) SNP-based heritability estimates for BD I and BD II stratified by cohort. (**d**) Genetic correlation estimates. The distributions of the 100 r_g-I/II_ and the 10 000 r_g-mix_ estimates are presented. A significant difference between the distributions was observed (*P*=6.25 × 10^−45^ for a two-sided *t*-test of mean difference between the empirical r_g-I/II_ and r_g-mix_ distributions). BD, bipolar disorder; BDRN, Bipolar Disorder Research Network; SNP, single-nucleotide polymorphism; SWEBIC, Swedish Bipolar Disorder Cohort.

We next compared BD subtypes with one another at the genetic level by estimating SNP-based heritability (SNP-*h*^2^)^[refs 30, 31, 32]^ with GCTA.^48^ Traditional estimates of heritability infer the total genetic contribution to the variance of a given phenotype through familial relationships. In contrast, SNP-based estimation has the advantage of harnessing actual genotype data, estimating the variance in liability to disease that can be attributed to SNPs across the whole genome. Reliable BD subtype SNP-*h*^2^ estimation required the ICCBD sample analyzed be balanced with respect to disease subtype and site of collection (see Materials and Methods). This was found to be the case when considering BD I and BD II in the SWEBIC and BDRN cohorts (2811 SWEBIC-BDRN BD I cases; 1398 SWEBIC-BDRN BD II cases; 11 164 SWEBIC-BDRN controls; Supplementary Tables 1–3). We observed a significant difference in the SNP-*h*^2^ of BD I and BD II (BD I SNP-*h*^2^=0.35, s.e.=0.02; BD II SNP-*h*^2^=0.25, s.e.=0.04; two-sided *t*-test *P*-value=0.02; Figure 3 and Supplementary Table 3).

We next estimated the genetic correlation between BD I and BD II (*r*_g__-I/II_)^[ref. 35]^ in SWEBIC–BDRN to directly assess their degree of genetic overlap. In general, this method requires that each case group be compared with an independent control group. In order to compare the BD I cases (*n*=2813) to the BD II (*n*=1397) cases in SWEBIC-BDRN, the 11 164 controls were therefore split into two groups. We estimated *r*_g__-I/II_ to be 0.78, the mean of 100 estimates calculated using 100 permutations of the splitting procedure used to create independent control groups (range=0.63–1.00, s.d.=0.07; see Materials and Methods; Figure 3). Positive *r*_g_ values are observed when case groups share the same risk alleles relative to controls. In principle, this value should equal one when the two case groups are random samples of the same case population. Indeed, when genetic correlations were estimated between case groups containing random mixtures of BD subtypes (*r*_g__-mix_; see Materials and Methods), a mean value of 0.97 was observed (range=0.64–1.00, s.d.=0.05). The distribution of *r*_g__-mix_ estimates was significantly higher than the distribution of *r*_g__-I/II_ estimates, suggesting the genetic heterogeneity observed between BD I and BD II is non-random and not simply a function of BD being a complex genetic disease (*P*=6.25 × 10^−45^ for *t*-test of mean difference between the empirical *r*_g__-I/II_ and *r*_g__-mix_ distributions; Figure 3).

### Polygenic scoring of BD subtypes

**Figure 4:**
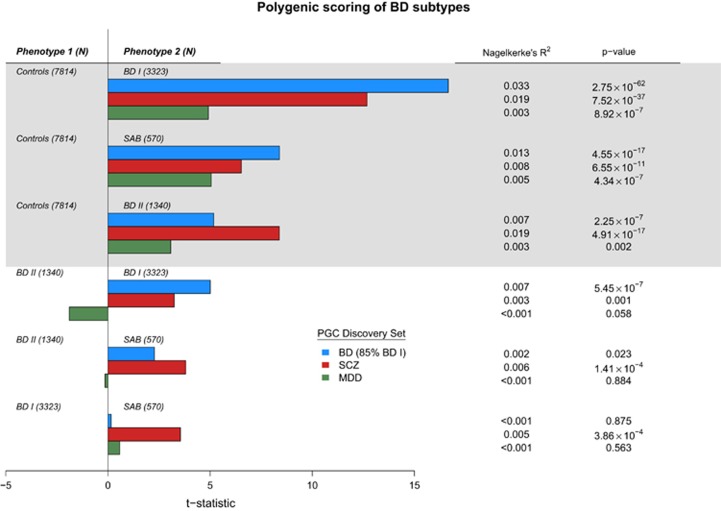
Comparison of BD subtypes with one another and controls using bipolar disorder (BD), schizophrenia (SCZ) and major depressive disorder (MDD) polygenic scores. Regression analyses of phenotype (top: BD subtype vs controls; bottom: BD subtype vs BD subtype) on polygenic scores derived from three previous GWAS (blue—BD, red—SCZ and green—MDD) were performed using MDS components and study site as covariates. The *t*-statistic plotted on the *x* axis is the ratio of the coefficient of the polygenic score variable and its standard error from the generalized linear model regression equation. The direction of the plotted bars indicates the phenotype in the comparison with the higher polygenic scores. The *P-*values for whether scores differed significantly between phenotypes are shown at the far right. Nagelkerke’s *R*^2^ values were derived from a corresponding logistic regression analysis performed for each phenotype comparison, and are used as to estimate the variance in phenotype explained by the polygenic score. GWAS, genome-wide association studies.

Polygenic scoring^29^ was also used to assess the relationships between disease subtypes (see Materials and Methods). Risk scores were derived for each individual in the data set by counting the number of alleles present that have previously shown to increase disease risk across a range of *P*-value thresholds. Logistic regression was performed between diagnosis and risk score with site and the first 10 MDS components as covariates to test for significant correlation between BD subtype and SCZ, MDD, and BD genetic risk. For all subtypes tested, each discovery set produced a significantly higher polygenic risk score for cases compared with controls, with the most significant correlation being that between BD risk scores and diagnosis of BD I (*P*=2.75 × 10^−62^, pseudo*-R*^2^=0.033; Figure 4). MDD risk scores were the least significant across all subtypes (BD I *P*=8.92 × 10^−7^, BD II *P*=4.34 × 10^−7^, SAB *P*=0.002). Next, we examined the ability of these risk scores to differentiate subtypes from each other. We again performed logistic regression on risk score except comparing BD subtypes to one another rather than to controls. BD risk scores had significant discriminant ability when comparing BD I and BD II, with more BD risk alleles found in BD I (*P*=5.45 × 10^−7^). In addition, we identified significantly more SCZ risk alleles in BD I compared to BD II (*P*=0.001), SAB compared with BD I (*P*=3.86 × 10^−4^), and SAB compared with BD II (*P*=1.41 × 10^−4^; Figure 4). MDD risk scores did not show significant discriminant ability between any pair of BD subtypes, though were higher in BD II compared to BD I (*P*=0.06) and in BD I compared with SAB (*P*=0.88; Figure 4).

## Discussion

We present a BD GWAS with over 5000 previously unreported cases (totaling 13 902 cases and 19 279 controls) identifying eight genome-wide significant loci, including a novel locus on chromosome 10. The new locus contains two coding genes, adducin 3 (*ADD3*) and aminopeptidase P (*XPNPEP1)* and a non-coding RNA also annotated as an antisense *ADD3* RNA. Both genes are biologically interesting in relation to BD. First, *ADD3* is a member of a family of cytoskeletal proteins responsible for capping the growing end of actin filaments and promoting the binding of spectrin to actin in the brain and elsewhere. Recently, adducins have been shown to form ladder-like structures in axons similar to those observed for actin and spectrin.^51^ Previously, genetic alterations in other components of the actin cytoskeleton have been suggested as risk factors for both BD and SCZ.^52^ Second, *XPNPEP1* is an X-prolyl aminopeptidase that mediates the proteolytic cleavage of the N-terminal amino acid in peptides with proline. Proline containing neuropeptides include oxytocin, corticotropin releasing hormone, neuropeptide Y and substance P,^53^ each of which has been implicated with varying levels of support in BD. Heterozygous mice in which *XPNPEP1* was deleted had smaller forebrains also consistent with a potential role in neuropsychiatric illness.^54^

Of the 12 loci previously reported genome-wide significant in at least 1 of the 3 largest BD GWAS, 9 have *P*-values below 10^−6^ in our data set and 7 of these surpass genome-wide significance (Supplementary Table 5). One straightforward explanation for our ability to support some, but not all prior BD loci, is the possibility of ‘winner’s curse’.^55^ Given a polygenic model, the power will be low to detect a particular variant (due to the overestimation of the power to replicate individual results) at genome-wide significant threshold, though there will be many chance opportunities to identify at least one variant.^55^ Thus any single lack of replication is not informative. In general, however, our data strongly support consistency of our samples with the prior literature as reflected by the sign test with the prior PGCBD samples (Supplementary Figure 4), significant polygenic risk prediction (Supplementary Figure 5) and BD heritability estimates (Supplementary Table 2) consistent with previous estimates.^30^

In this paper, we provide evidence of a molecular correlate for the division of BD into types I and II. SNP-*h*^2^ was observed to be significantly higher in BD I compared with BD II. This observation led us to test the null hypothesis that BD I and BD II are the same genetic trait (that is, *r*_g__, I/II_=*r*_g__, mix_). The mean *r*_g__, I/II_ and *r*_g__, mix_ values were significantly different from one another, supporting the notion that BD I and BD II cohorts have a significantly lower genetic correlation than two BD cohorts composed of both subtypes. In addition to SNP-*h*^2^ and *r*_g_ analyses, we found that polygenic score profiles of BD and SCZ risk variants had significant discriminant ability between BD I and BD II. The BD risk variants used for scoring were originally discovered in a cohort that is 85% BD I and 11% BD II,^24^ thus the lower scores in the ICCBD BD II cohort is consistent with BD II having partially distinct risk loci. The discriminant ability of SCZ risk variants with higher scores in BD I is consistent with clinical phenomenology, as psychosis is far more commonly a feature of BD I than BD II.^56^ We hypothesized that MDD risk scores would be significantly higher in BD II compared with BD I due to the prominent depressive episodes required for a diagnosis of BD II. This was not found to be the case, though a non-significant trend in the predicted direction was observed.

The number of SAB cases in the ICCBD cohort allowed us to begin to assess its genetic overlap with BD I and BD II. Subtype-specific association results for the eight loci identified as significant in the ICCBD–PGCBD GWAS showed the expected direction of effect for all three subtypes at seven loci and for two of the three subtypes (BD I and BD II) at one locus (Supplementary Table 6). Thus, for these eight most significant loci, the association does not appear to be driven by a particular subtype of disease. Clinically, SAB is unique among BD subtypes in that psychosis in the absence of mania and depression is required for a diagnosis. As such, one may expect that the psychosis risk loci from studies of SCZ would demonstrate discriminant ability between SAB and other BD subtypes, which is indeed what we observe. We see a pattern of SCZ risk loci loading that follows the prominence of psychotic symptoms in the phenomenology of the three BD subtypes (SAB>BD I>BD II). One interpretation of these findings is that the genetic liability of psychosis influences the prominence of psychotic symptoms across diagnostic entities, ranging from psychosis being the sole feature and occurring only in the absence of mania or depression (that is, SCZ), to being a feature occurring in both the presence and absence of mania or depression (that is, SA), to being a common but not required feature occurring only in the presence of mania or depression (that is, BD I), to finally being a feature occurring rarely and only in the presence of depression (that is, BD II and MDD). However, it should be noted that the SCZ risk variants used for scoring were discovered in a dataset (PGCSCZ^45^) that included SA cases. Of the 5 PGCSCZ cohorts that included SA, three explicitly reported the percentage of SA cases, which ranged from 6 to 17% of the total cohort sample (the percent of these cases with SAB is unavailable).^45^ Therefore, the increased SCZ loading in SAB observed here could in part reflect genetic signal from SAB cases in the PGCSCZ discovery set in addition to (or, as opposed to) reflecting an increased loading for risk variants specific to psychosis. In order to further address these possibilities through genetics, larger SAB cohorts such as those that will be available in larger consortia are necessary.

This study has several limitations. Although our sample sizes for BD subtype comparisons are larger than those previously reported, we were still relatively underpowered to perform subtype-specific GWAS. As is common for most large-scale genetic studies in psychiatry, we were not able to assess higher-dimensional phenotypes (that is, age of onset, lithium response) since this information is not currently available for all ICCBD subjects. Finally, heterogeneity between sites was observed in our analyses, most notably in that the GPC cohort demonstrated relatively decreased signal compared with the SWEBIC and BDRN cohorts. Such heterogeneity is not uncommon in genetic studies that pool samples from many different sites, but also raises the possibility that technical artifacts or subtle confounders (that is, cryptic relatedness) unable to be detected using current methods may be blunting the signal. When possible, the potential for such batch effects was taken into account (Supplementary Note).

In summary, in addition to providing a new and interesting locus for BD, our findings begin the process of correlating molecular signatures with disorders on the bipolar spectrum of mental illness. Increasingly, it is realized that there is genetic overlap between psychiatric diagnoses. Here we add nuance to our understanding of this phenomenon, providing evidence that genetic overlap correlates with overlap in clinical phenomenology. Ultimately, these analyses could lead to refined distinctions with the potential to improve prognosis and treatment strategies. Whether there are other dimensional measures that may better reflect the underlying genetic architecture remains to be tested in larger consortia samples as increasingly refined phenotyping is available.
