# Supplementary material for: Evidence for genetic heterogeneity between clinical subtypes of bipolar disorder
Source: Transl Psychiatry. 2017 Jan 10;7(1):e993–. doi: 10.1038/tp.2016.242 (PMC5545718; doi:10.1038/tp.2016.242)
Supplement: Supplementary Material [file tp2016242x1.pdf]

Supplementary Material for

Evidence of genetic heterogeneity between subtypes of bipolar disorder

Table of Contents

Phenotyping 3
Swedish Bipolar Cohort (SWEBIC) 3
Bipolar Disorder Research Network (BDRN) 3
Genomic Psychiatry Consortium (GPC) 3
Inter-site phenotypic comparisons 3

Genotyping 3
SWEBIC 3
BDRN 3
GPC 4

Quality Control 4
SWEBIC 4
BDRN 4
GPC 4

Assessing for potential source of diminished BD signal in GPC cohort 54

Supplementary Figures 6
Supplementary Figure 1: Multidimensional scaling (MDS) plots. 6
Supplementary Figure 2: Quantile-quantile (QQ) plots. 7
Supplementary Figure 3: Forest and regional association plots. 8
rs56361249 (ICCBD-PGCBD) 9
rs2302417 (ICCBD-PGCBD) 11
rs1203233 (ICCBD-PGCBD) 12
Chromosome 9 indel (ICCBD) 13
rs10994299 (ICCBD-PGCBD) 14
rs10884920 (ICCBD) 15
rs10884920 (ICCBD-PGCBD) 16
rs4765913 (ICCBD-PGCBD) 17
rs10459221 (ICCBD-PGCBD) 18
Supplementary Figure 4: Sign test for ICCBD using PGCBD discovery. 19
Supplementary Figure 5: Polygenic scoring ICCBD with PGCBD discovery. 20

Supplementary Tables 21
Supplementary Table 1: Sample characteristics. 21
Supplementary Table 2: Heritability estimates by site. 22
Supplementary Table 3: Heritability and genetic correlation estimates of BD I and BD II 23
Supplementary Table 4: Genome-wide significant loci in the ICCBD GWAS. 24
Supplementary Table 5: Previously identified BD GWAS loci in ICCBD-PGCBD. 25
Supplementary Table 6: ICCBD-PGCBD loci by BD subtype. 26

References 27

Phenotyping

Swedish Bipolar Cohort (SWEBIC)

Swedish National Quality Assurance Registry (QAR). The QAR was developed in 2004 in order to capture basic clinical epidemiological data of BD along with longitudinal data on the natural history and clinical course of the disease, as well as to improve the overall quality of the care of BD patients in Sweden. Inclusion in the QAR requires a diagnosis of BD I or II and age > 18. In order to validate the QAR data, the Structured Clinical Interview for DSM-IV[1] (SCID) affective module was conducted on a randomly selected sub-sample (5%) living in the Stockholm area by board-certified psychiatrists.
St. Goran. The diagnostic instrument used for these subjects was a Swedish adaptation of the Affective Disorder Evaluation[2] which includes the affective module of the SCID[1].

Bipolar Disorder Research Network (BDRN)

Systematic recruitment was conducted via the UK National Health Service (NHS) Mental Health Research Network. NHS nurses and psychologists identified suitable participants from community mental health teams and other secondary care clinics, then obtained informead consent from these participants. Non-systematic recruitment involved advertising for participants via local and national media and enlisting the help of self-help organizations. A subset of the BDRN cases have been reported by Green et al[3] in a study of 3,106 single-nucleotide polymorphisms (SNPs) previously implicated in BD.

Genomic Psychiatry Consortium (GPC)

A comprehensive description of the collection procedures for the GPC cohort has been previously published[4].

Inter-site phenotypic comparisons

We established a Phenotype Committee including at least 1 trained clinician from each participating site in order to assess the comparability of phenotypic classification across ICCBD cohorts. Each site contributed a set of notes from cases and from individuals that did not meet criteria for Bipolar Disorder but did meet criteria for related mood or psychotic disorders (also known as distractors). The notes were compiled in such a way as to keep them blinded with respect to case/distractor status. Each record included the full de-identified and finalized set of diagnostic data that were used by the sites' trained clinicians to evaluate diagnosis. Each of the Phenotype Committee members provided independent ratings of the primary variable (case vs distractor) by reviewing the records. A quantitative analysis was conducted to determine the degree to which the committee members agree with diagnoses made by the trained clinicians. The inter-rater reliability was assessed using Fleiss' Kappa statistic for multiple raters ($\kappa = 0.72$ for the primary diagnostic variable).

Genotyping

SWEBIC

DNA extraction occurred at the Karolinka Institutet. Samples were genotyped in 5 batches (denoted Wave2-Wave6) using Affymetrix 6.0 (Wave2-Wave4; 40.1% of SWEBIC cases, 37.4% of SWEBIC controls) and Illumina OmniExpress (Wave5-Wave6; 59.9% of SWEBIC cases, 62.6% of SWEBIC controls) chips according to the manufacturers' protocols. Reported elsewhere is data from Wave2-Wave4 controls [2,5,6], Wave4 cases[2], and Wave5 controls[6].

BDRN

Case subjects DNA extraction occurred at the neuropsychiatric genetics laboratory at Cardiff University. Samples were genotyped using two platforms: 1520 cases were genotyped using Illumina OmniExpress, and 1128 cases were genotyped using the Illumina ComboChip. BDRN control subjects were genotyped using the Illumina 1.2M Custom Chip designed for the WTCCC GWAS studies, as described elsewhere[7].

GPC

Samples were genotyped at the Broad Institute on the Illumina OmniExpress Chip following standard protocols.

Quality Control

SWEBIC

The SWEBIC Affymetrix data was obtained in two batches ***(Supplementary Table 1).*** The first batch consisted of controls from Wave2 and Wave3. The second batch consisted of Wave4 cases and controls. Each batch passed through our standard QC pipeline. After all of the initial QC checks, the SWEBIC Affymetrix datasets were merged, and this combined dataset underwent another iteration of QC. The SWEBIC Illumina data was also obtained in two batches. The first batch consisted of controls from Wave5. The second batch consisted of Wave6 cases and controls. After each of these batches was run through the QC pipeline, the SWEBIC Illumina datasets were merged and the merged set underwent another iteration of QC. Next, the SWEBIC Affymetrix and SWEBIC Illumina datasets were merged to evaluate further for population stratification within the SWEBIC ICCBD. After all QC, 923 cases and 2,215 controls remained in the SWEBIC Affymetrix dataset and 1,378 cases and 3,716 controls remained in the SWEBIC Illumina dataset.

BDRN

Initially four batches passed through the QC pipeline: 2 case batches and 2 control batches. After these initial QC checks, the two case batches were merged, and the two control batches were merged. Each of the merged sets was then passed through the QC pipeline. Finally, the cases were merged with the controls and a third iteration of QC was performed. After QC, 2,609 cases and 5,413 controls remained in the BDRN cohort. Prior to performing the ICCBD-PGCBD meta-analysis it was necessary to identify BDRN samples that were either duplicates or relatives of individuals in the PGCBD study. To identify such overlaps, the PGCBD datasets were obtained and merged with the BDRN data. Identity-by-state analyses were performed and pairs of individuals related at a PIHAT value > 0.1 were identified. These 2,610 controls and 26 cases were removed from the ICCBD prior to the ICCBD-PGCBD meta-analysis. As such, the final meta-analysis contained only unique individuals, with each individual belonging to exactly one sample.

GPC

The USC data was obtained in a single batch and passed through the QC pipeline, leaving 1,537 cases and 1,295 controls with high quality genotyping data.

Controlling for potential batch effects

The ICCBD study design leaves open the possibility for batching to occur at various levels, including study site (e.g., differences between SWEBIC, BDRN and GPC), genotyping platforms (e.g., differences between SWEBIC Illumina and SWEBIC Affymetrix) and genotyping waves (e.g., differences between SWEBIC Affymetrix Wave 1, SWEBIC Affymetrix Wave 2, etc.). This is a consequence of performing large genetic studies that require sample sizes exceeding those that can be attained through a single research center. As a result, much effort has been made over the past decade to arrive at “best practices” for reducing the effects of these design limitations, which were utilized here. In order to account for genotyping platform batch effects, we followed a common practice in GWAS, namely, to impute and analyze each dataset separately, then meta-analyze results across studies using standard approaches. In this case, we analyzed 4 datasets individually (SWEBIC-Affy, SWEBIC-Illu, BDRN and GPC), then performed a meta-analysis of the results. This approach, compared to pooling samples and analyzing as a single large dataset using genotyping platform as a covariate (so-called “mega-analysis”), in principle reduces the likelihood of false positives resulting from population stratification or genotyping platform. The SWEBIC-Affy, SWEBIC-Illu, BDRN and GPC analyses performed prior to meta-analysis all utilized MDS components are covariates, but since each analysis included only a single genotyping platform it was not necessary to include this as a covariate. As an additional quality control measure, SNPs in the genome-wide significant loci identified in the ICCBD-PGCBD were “mega-analyzed” in

the manner described above using site as a covariate, and all remained genome-wide significant when analyzed in this manner.

Assessing for potential source of diminished BD signal in GPC cohort

We observed diminished genetic signal in the GPC cohort compared to SWEBIC and BDRN as measured by sign tests, polygenic scores and SNP-h^2 estimates (although the sign tests and polygenic scores were significant in the expected direction; ***Supplementary Figures 4 and 5***). A suite of analyses beyond those performed in the standard QC pipeline were therefore conducted to further clarify whether this was due to technical artifact or simply consistent with the known inter-site variability that occurs when datasets are analyzed in combination for large international genetic studies[8]. Phenotyping error, batch effects, and population substructure were all investigated as possible sources of the blunted GPC signal. To rule out phenotype error as a source of the diminished GPC signal, after all analyses were completed an expert diagnostician went back to perform a manual review of participant charts. Sufficient chart information was available for all GPC subjects included in the primary ICCBD GWAS and in all instances the phenotype (case or control) utilized in the analysis was confirmed by the manual chart review. To further investigate potential phenotype error, a more restrictive group of GPC cases was created using the following criteria: high OPCRIT ratings for items suggestive of severe illness, positive family history of psychiatric illness, a history of psychiatric hospitalization, no history of substance abuse and no history of seizures. Polygenic scores were re-analyzed after applying these filters and no major differences were observed compared to such analyses performed on the full GPC dataset (data not shown). To determine if a technical artifact was contributing to the decreased GPC signal, polygenic scores were re-analyzed according to (a) genotyping plate and (b) site of enrollment. No batch effects were identified (data not shown). To investigate the possibility that population substructure could be contributing to the low GPC SNP-h^2 estimates, these values were re-calculated without MDS covariates. The resulting estimates were, indeed, more consistent with those expected for BD (data not shown). We therefore performed strict principal component matching and relatedness filtering and re-calculated SNP-h^2, but these steps did not modify account for the decreased signal. The GPC cohort reported in this manuscript is the European subset of a multiethnic cohort (determined by both self-identified ethnicity and PCA). Two equally sized clusters (Northern and Southern European) of both cases and controls are observed.

Supplementary Figures

Supplementary Figure 1: Multidimensional scaling (MDS) plots.

MDS plots for the individuals in the final post-QC ICCBD dataset (top panel), as well as the SWEBIC, BDRN and GPC study samples (bottom panel). Cases are indicated in red, controls in blue.

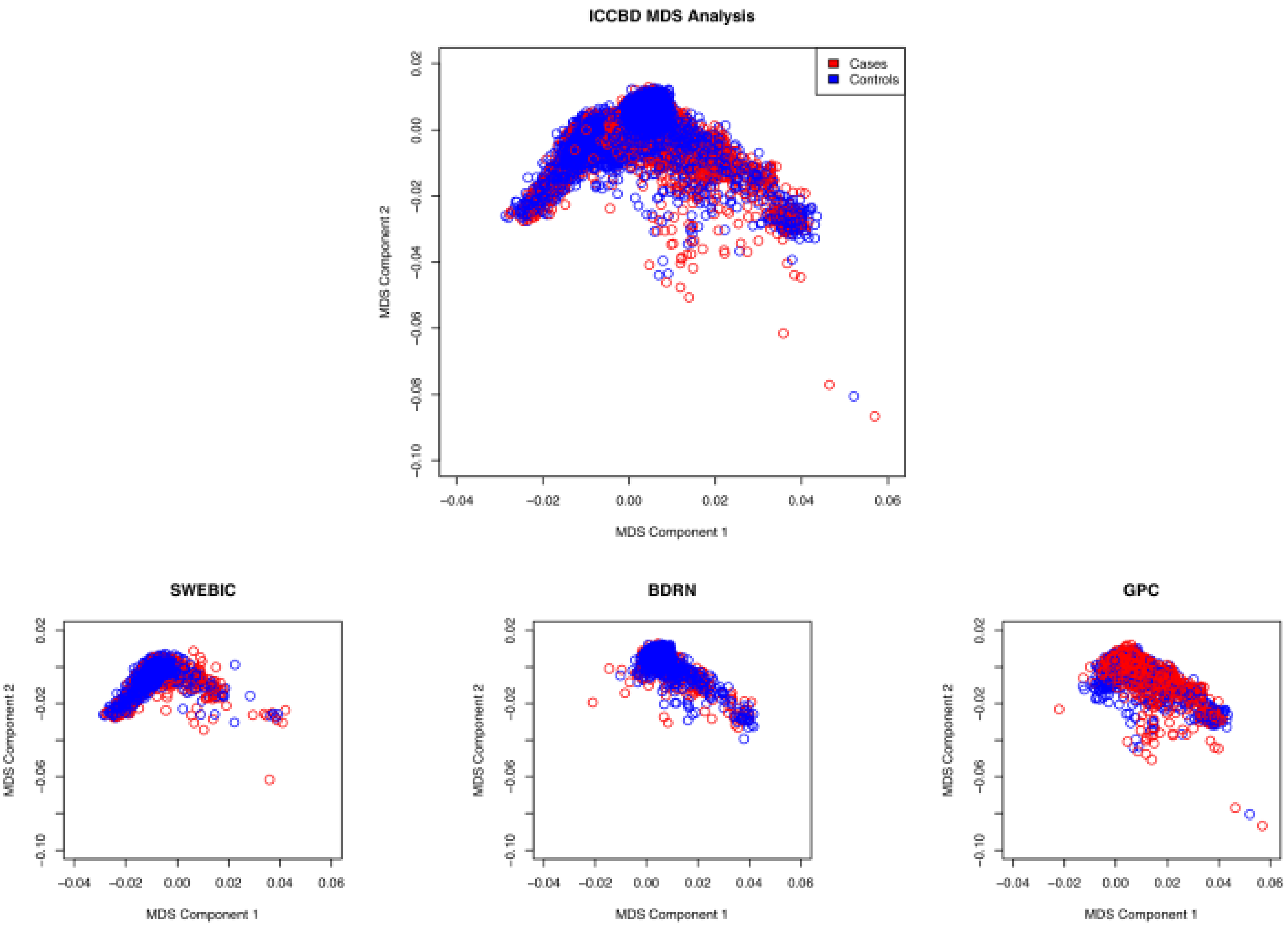

Supplementary Figure 2: Quantile-quantile (QQ) plots.

QQ plots of single SNP statistics based on SNP dosage for ICCBD (red) and PGCBD (black) GWAS. Covariates for MDS and site were included for all tests. Genomic inflation factor (λ) is 1.11 for ICCBD (1.03 in SWEBIC Affymetrix, 1.05 in SWEBIC Illumina, 1.08 in BDRN and 1.01 in GPC; see table in Figure 1) and 1.13 for PGCBD.

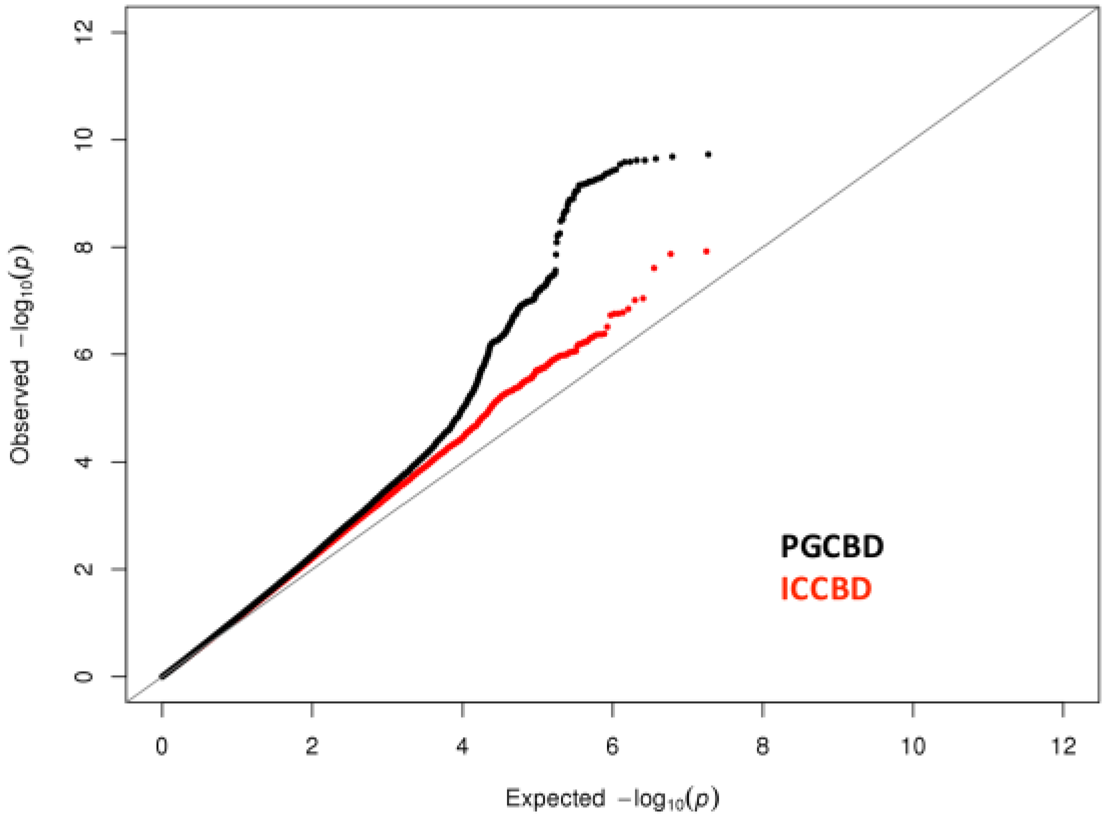

Supplementary Figure 3: Forest and regional association plots.

Regional p-value and forest plots for each of the 2 regions achieving genome-wide significance ($P < 5 \times 10^{-8}$) in the ICCBD GWAS and the 8 regions achieving genome-wide significance in the ICCBD-PGCBD meta-analysis. Each of the following 10 pages has one of these 9 regions (one region was significant in both ICCBD alone and the ICCBD-PGCBD meta-analysis and thus has 2 pages). The index SNP and the dataset (ICCBD or ICCBD-PGCBD) from which the regional plot is derived appears in the page title. Of note, for the ICCBD GWAS in some instances there is a discrepancy between the *P* value in the forest plot and that in the regional plot; this is not an error, but rather is due to the forest plot *P* values being generated from combining the genotype data for the 4 ICCBD sites into a single dataset that was then analyzed for association. This approach allows for the calculation of allele frequencies, OR and SE in the table. In contrast, the regional plot *P* values are derived from meta-analyzing the 4 ICCBD sites.

Forest plots (top panel of each of the following 10 pages). Depicted are the results for the SNP in the title in each ICCDBD site, the ICCBD combined GWAS, the PGCBD GWAS and the ICCBD-PGCBD meta-analysis. info = imputation quality score; f_case = frequency in cases; f_ctrl = frequency in controls; OR = odds ratio; SE = standard error of the odds ratio; het_P = heterozygosity *P* value; het_I = I^2 heterogeneity index; SWEBIC_af = SWEBIC sample genotyped on the Affymetrix platform; SWEBIC_il = SWEBIC sample genotyped on the Illumina platform.

Regional plots (bottom panel of each of the following 10 pages). The x-axis is chromosomal position (in kb) and the y-axis is significance of association represented as $-\log_{10}(P)$. The dotted gray line shows the genome-wide significance level (5×10^{-8}). The index SNP is marked by the large red diamond, its name and *P* value nearby. Dot color is proportional to LD between the plotted SNP and the index as represented by r^2. Legend for r^2 is given in upper right corner. Green lines in lower half represent genes with black vertical lines for exons. The blue line denotes regional recombination rates derived from HapMap.

rs56361249 (ICCBD-PGCBD)

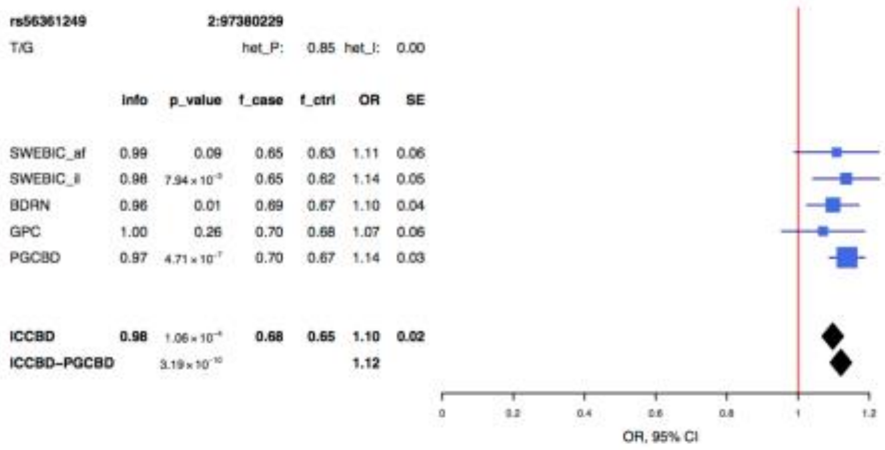

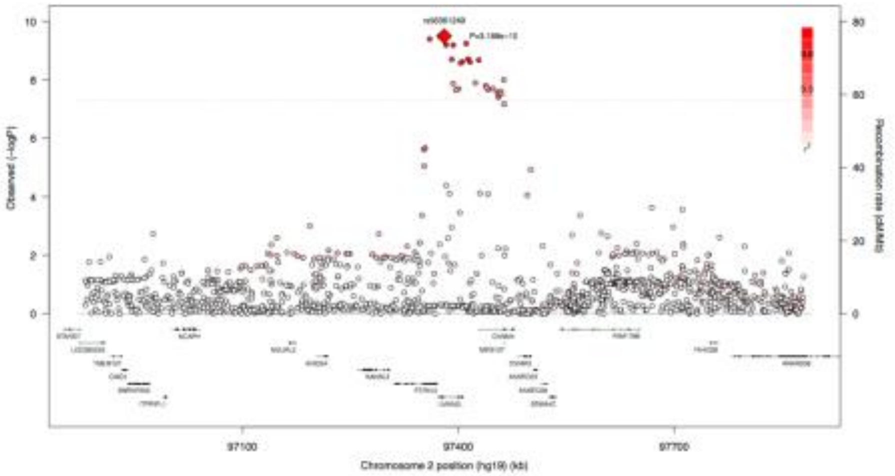

rs9834970 (ICCBD-PGCBD)

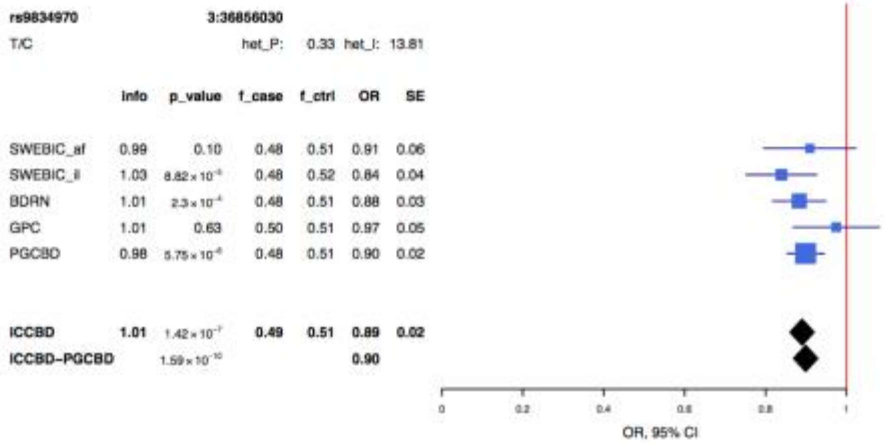

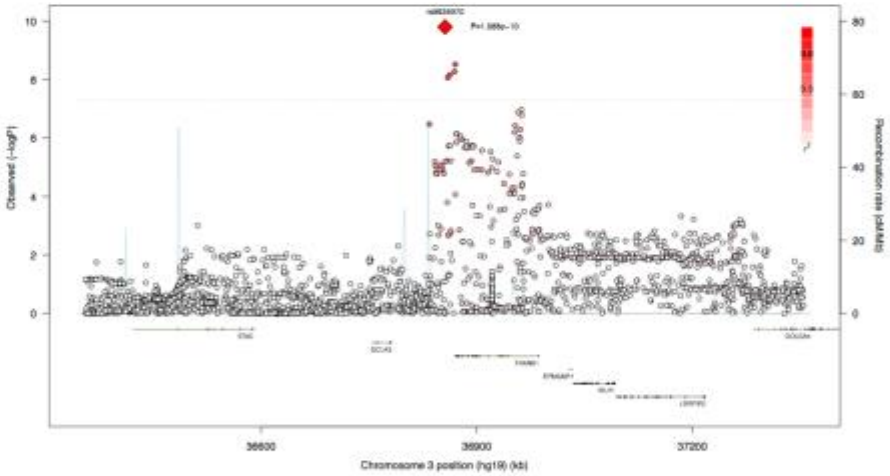

rs2302417 (ICCBD-PGCBD)

rs2302417		3:52814256				
T/A			het_P:	0.68	het_I:	0.00
	**info**	**p_value**	**f_case**	**f_ctrl**	**OR**	**SE**
SWEBIC_af	0.99	0.70	0.51	0.51	1.02	0.06
SWEBIC_II	1.00	0.06	0.52	0.50	1.09	0.05
BDRN	0.97	1.63×10^{-3}	0.55	0.52	1.12	0.03
GPC	0.95	0.03	0.54	0.50	1.13	0.06
PGCBD	0.97	2.8×10^{-6}	0.46	0.48	0.89	0.02
**ICCBD**	**0.98**	3.16×10^{-5}	**0.54**	**0.51**	**1.10**	**0.02**
**ICCBD–PGCBD**		2.75×10^{-8}			**1.11**	

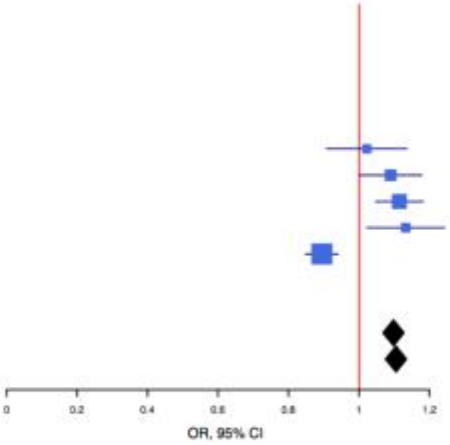

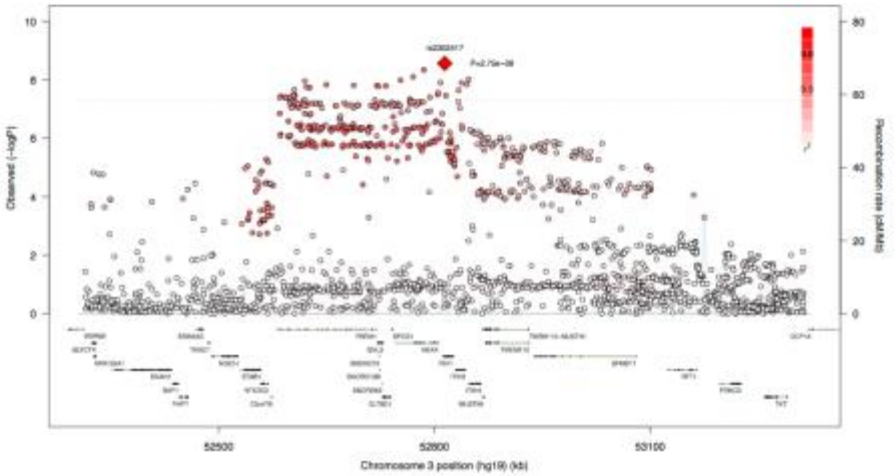

rs1203233 (ICCBD-PGCBD)

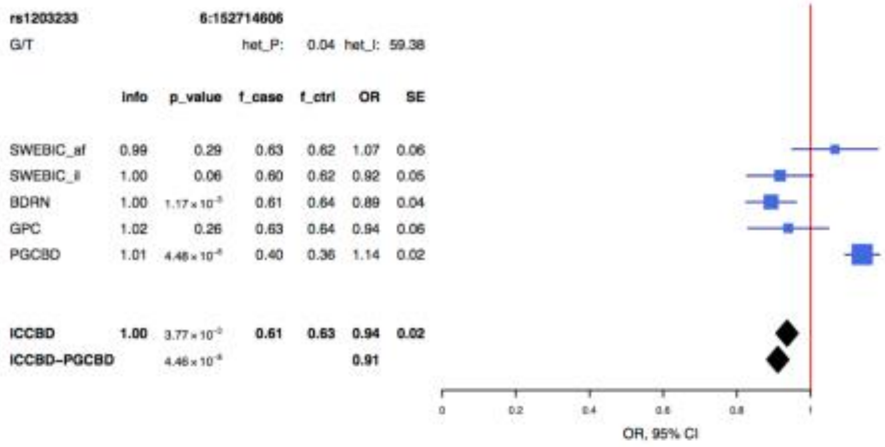

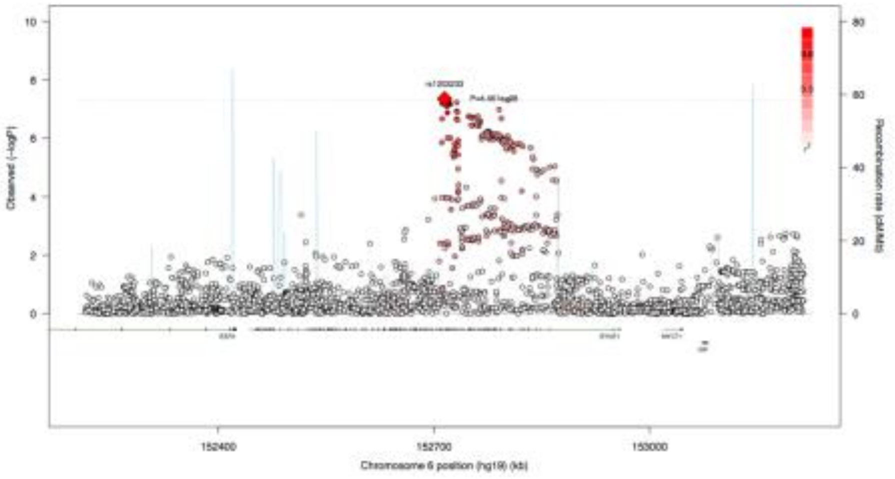

Chromosome 9 indel (ICCBD)

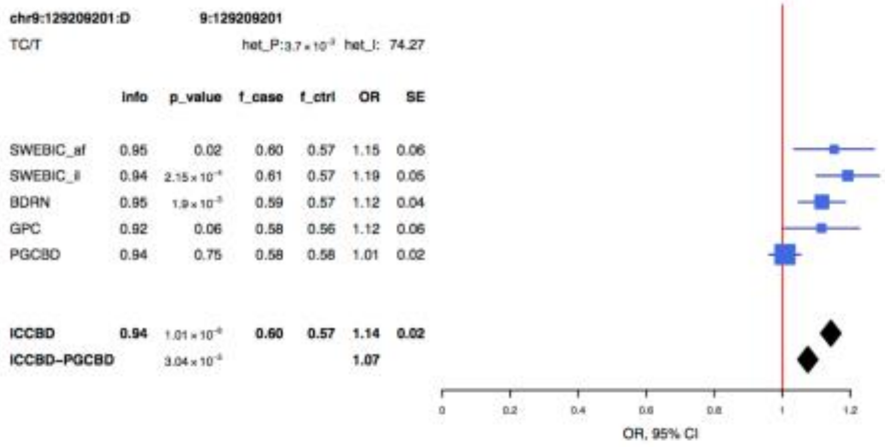

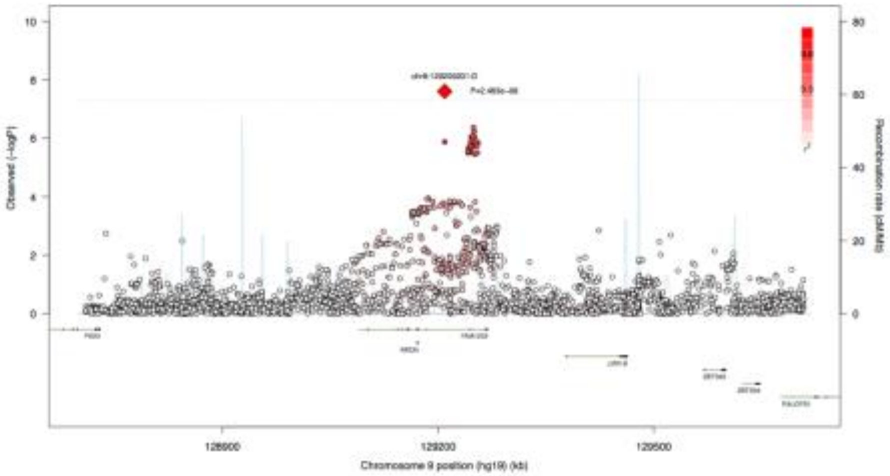

rs10994299 (ICCBD-PGCBD)

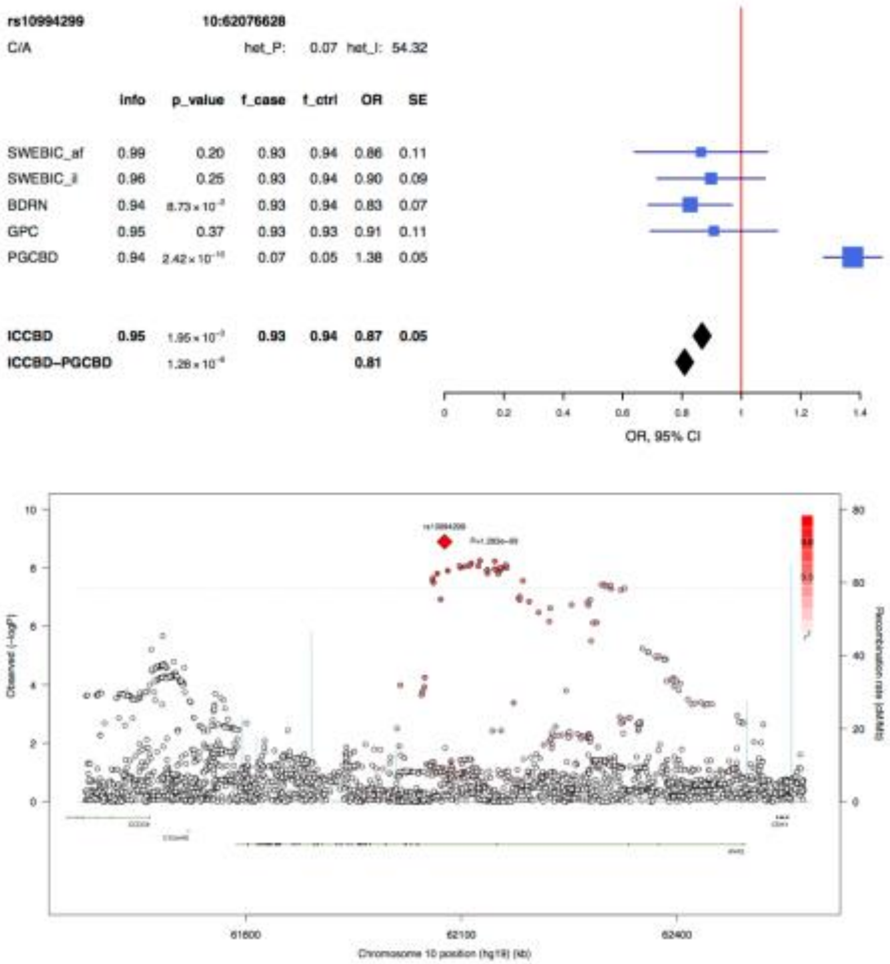

rs10884920 (ICCBD)

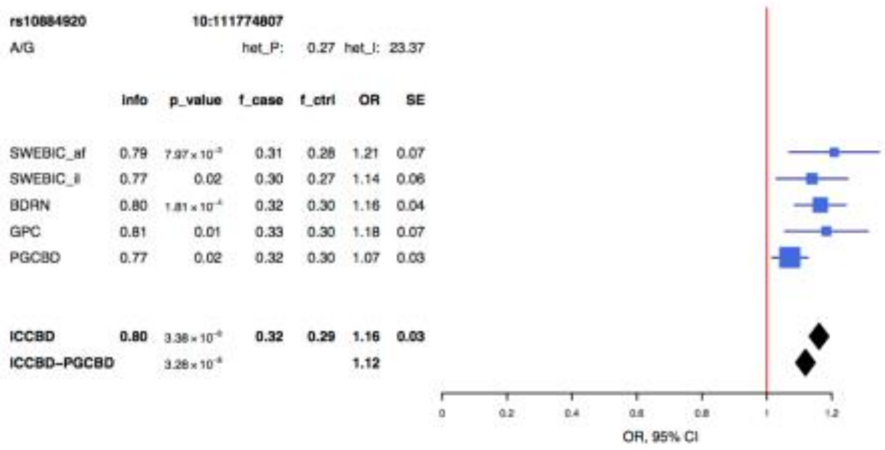

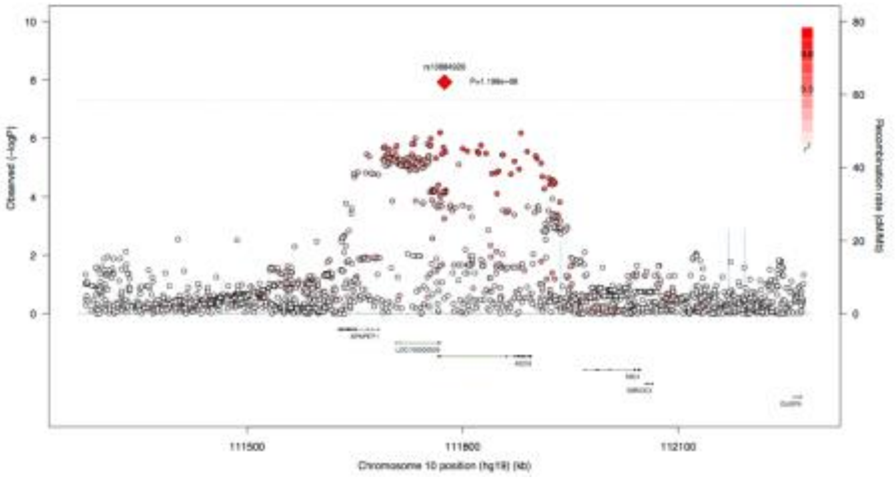

rs10884920 (ICCBD-PGCBD)

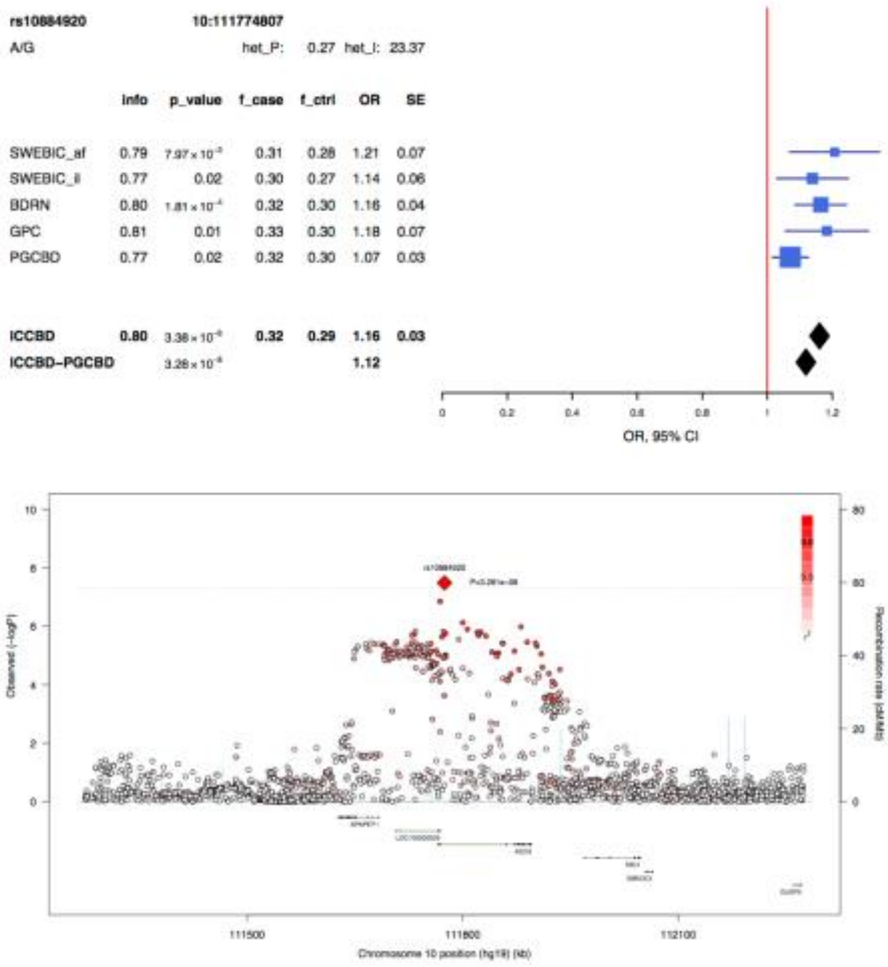

rs4765913 (ICCBD-PGCBD)

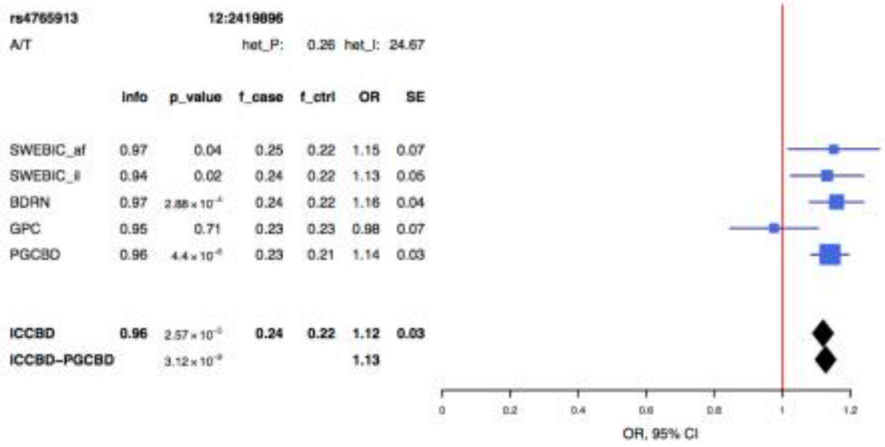

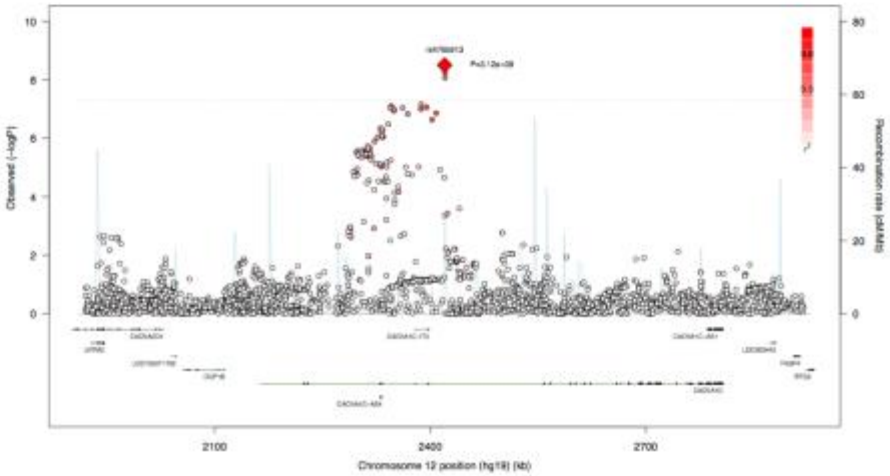

rs10459221 (ICCBD-PGCBD)

rs10459221		12:49466994				
C/G			het_P:	0.02	het_I:	64.48
	info	p_value	f_case	f_ctrl	OR	SE
SWEBIC_af	0.98	1.55 × 10^[illegible]	0.53	0.58	0.83	0.06
SWEBIC_II	0.99	0.81	0.57	0.57	1.01	0.05
BDRN	1.00	0.02	0.56	0.58	0.93	0.03
GPC	0.98	0.21	0.57	0.58	0.93	0.06
PGCBD	0.97	6.98 × 10^[illegible]	0.55	0.57	0.88	0.02
**ICCBD**	**0.99**	1.58 × 10^[illegible]	**0.56**	**0.57**	**0.93**	**0.02**
**ICCBD–PGCBD**		2.33×10^{-8}			**0.91**	

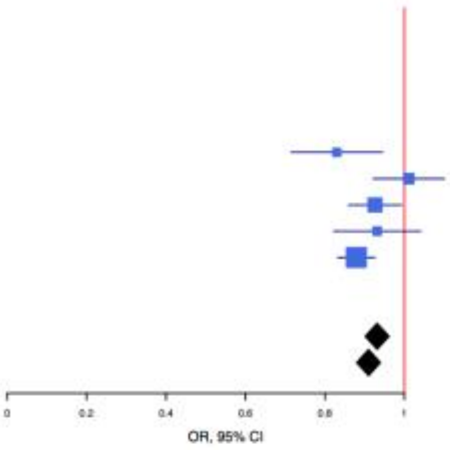

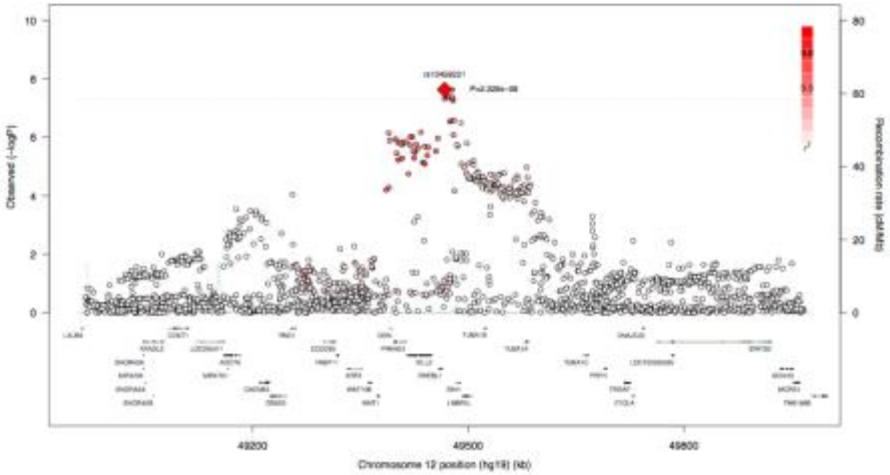

Supplementary Figure 4: Sign test for ICCBD using PGCBD discovery.

SNPs in the ICCBD found to be associated with BD below four p-value thresholds in the PGCBD analysis were evaluated. The fraction of SNPs with odds ratio in the ICCBD in the same direction as that in the PGCBD is plotted on the y-axis. The number of SNPs used for the *P* thresholds plotted from left to right on the x-axis were 1,029, 189, 31, and 8. The *P* values are indicated only for those tests that were significant below 0.05.

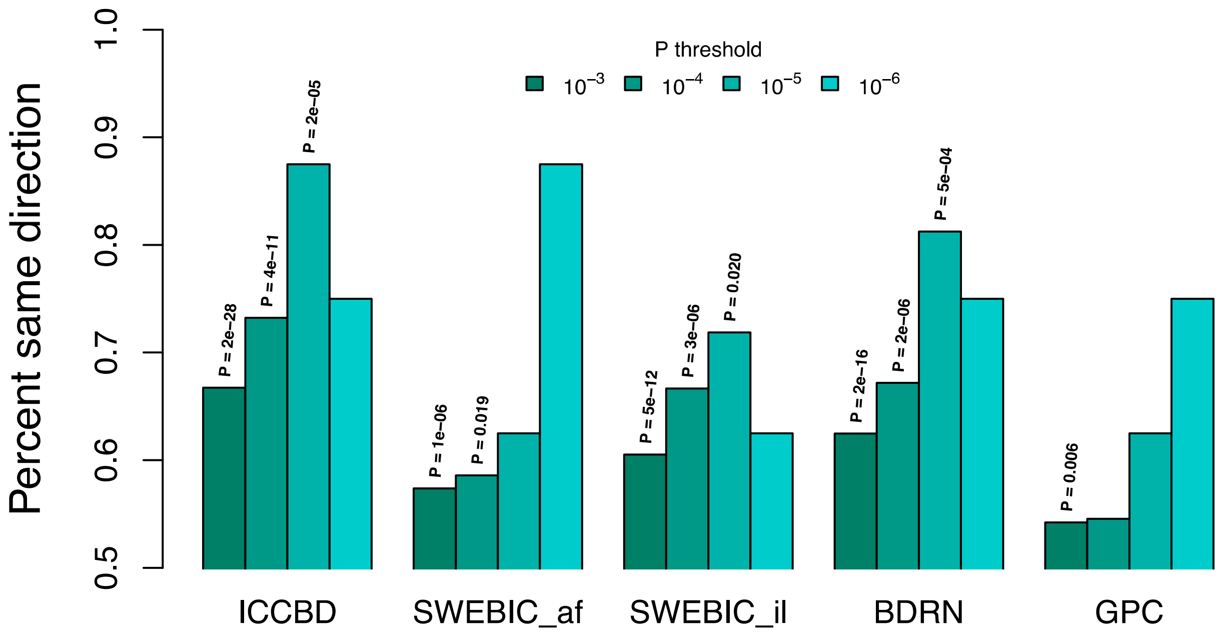

Supplementary Figure 5: Polygenic scoring ICCBD with PGCBD discovery.

Scores were assessed in the full ICCBD sample as well as within each cohort using the full PGCBD discovery set. The x-axis shows the discovery P threshold and the y-axis shows the Nagelkerke's R^2 value. *P* values are indicated above each bar for the corresponding test.

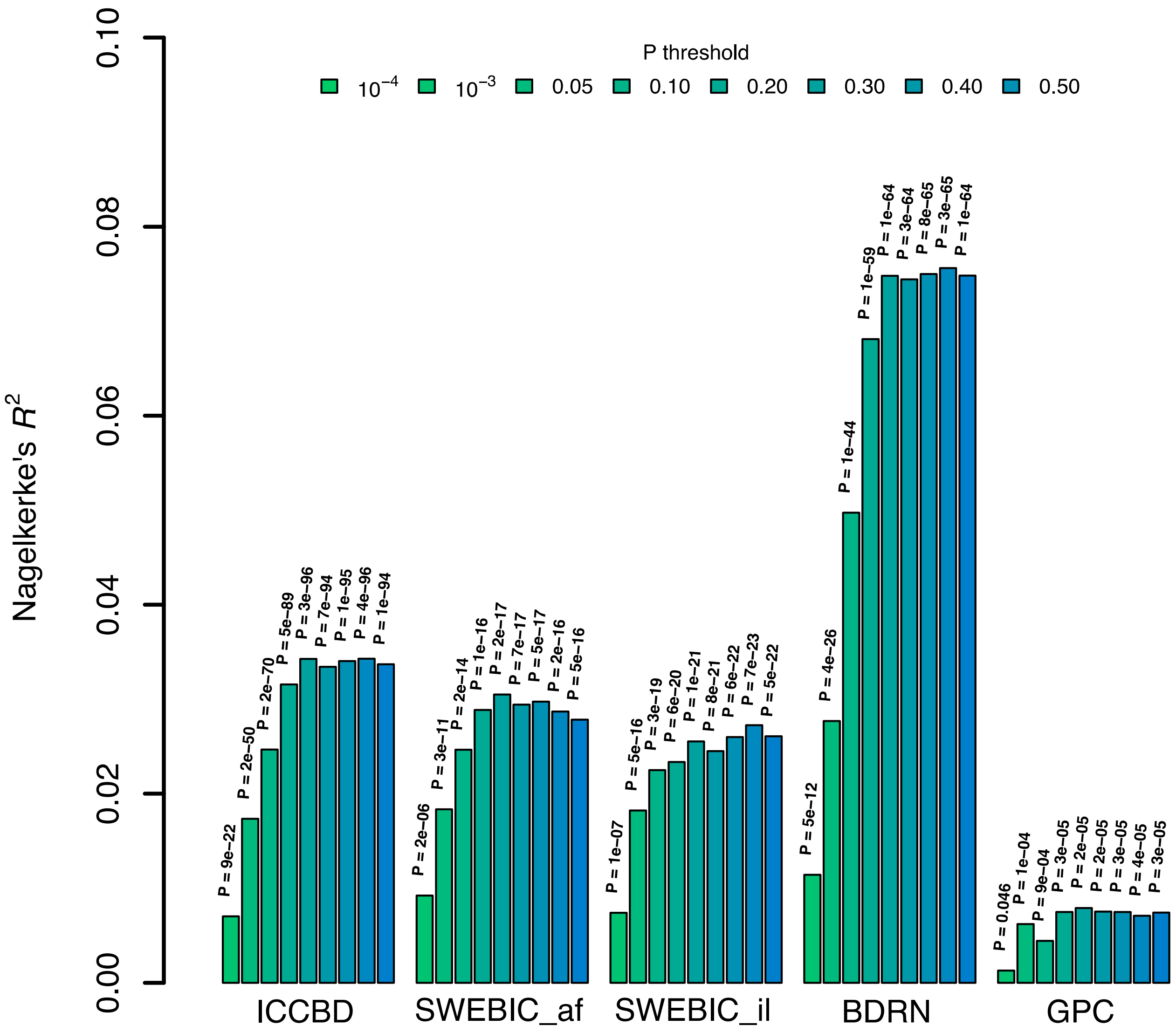

Supplementary Tables

Supplementary Table 1: Sample characteristics.

Number of samples for each ICCBD cohort for the GWAS, heritability and polygenic scoring analyses reported in the manuscript.

Cohort	Ethnicity	Chip	Batch [a]	GWAS						Heritability [b]						Polygenic scoring of BD subtypes					
				Cases	Controls	BD I	BD II	SAB	NOS	Cases	Controls	BD1	BD2	SAB	NOS	Cases	Controls	BD I	BD II	SAB	NOS
SWEBIC	European	Affymetrix 6.0	Wave2	0	242	0	0	0	0	0	239	0	0	0	0	0	0	0	0	0	0
		Affymetrix 6.0	Wave3	0	794	0	0	0	0	0	776	0	0	0	0	0	0	0	0	0	0
		Affymetrix 6.0	Wave4	923	1,179	543	114	20	246	916	1,170	540	114	20	242	0	0	0	0	0	0
		Omni Express	Wave5	0	2,552	0	0	0	0	0	2,464	0	0	0	0	0	2,552	0	0	0	0
		Omni Express	Wave6	1,378	1,164	607	508	30	233	1,335	1,131	591	492	30	222	1,378	1,164	607	508	30	0
BDRN	European	Combo Chip	-	1,104	0	689	369	34	12	1,103	0	688	369	34	12	1,083	0	681	369	33	0
		Omni Express	-	1,505	0	998	428	59	20	1,487	0	985	424	59	19	1,468	0	984	425	59	0
		Illumina 1.2M	58BC	0	2,805	0	0	0	0	0	2,794	0	0	0	0	0	1,469	0	0	0	0
		Illumina 1.2M	NBS	0	2,608	0	0	0	0	0	2,586	0	0	0	0	0	1,334	0	0	0	0
GPC	European-American	Omni Express	-	1,537	1,295	1,051	38	448	0	1,445	1,190	987	38	420	0	1,537	1,295	1,051	38	448	0
**ICCBD**				**6,447**	**12,639**	**3,888**	**1,457**	**591**	**511**	**6,286**	**12,350**	**3,791**	**1,437**	**563**	**495**	**5,466**	**7,814**	**3,323**	**1,340**	**570**	**0**

[a] GWAS analyses including Swedish samples from waves 2-4 have been previously reported in Bergen et al (reference 30). SWEBIC controls from all waves have been reported in Ripke et al (reference 36). BDRN controls are from the Wellcome Trust Case Control Consortium (reference 37).

[b] Analyses reported in Supplementary Table 2 include all BD subtypes from all ICCBD sites, whereas those in Figure 3 and Supplementary Table 3 include only BD I and BD II in SWEBIC and BDRN. The numbers of cases/controls for individual sites may differ from those in Supplementary Tables 2 and 3 due to cryptic relatedness filters being applied at different levels of resolution (i.e., across sites or within sites).

Supplementary Table 2: Heritability estimates by site.

SNP-h^2 of BD and subtypes for each ICCBD site and the combined dataset. The number of cases/controls listed in the N columns accounts for removal of individuals with cryptic relatedness > 0.05. The full set of site controls was used for each subphenotype calculation. Case/control counts for individual sites may differ from those in Supplementary Tables 1 and 3 due to cryptic relatedness filters being applied at different levels of resolution (i.e., across sites or within sites).

Site	BD			BD-I			BD-II			SAB		
	N	h^2	SE	N	h^2	SE	N	h^2	SE	N	h^2	SE
SWEBIC	2,257/5,782	0.28	0.03	1,135/5,782	0.40	0.05	607/5,782	0.23	0.08	50/5,782	0.87	0.85
BDRN	2,595/5,384	0.31	0.03	1,678/5,384	0.37	0.04	793/5,384	0.28	0.07	93/5,384	1.12	0.48
GPC	1,449/1,193	0.06	0.07	992/1,193	0.00	0.08	37/1,193	1.83	1.22	420/1,193	0.19	0.14
ICCBD	6,286/12,350	0.24	0.01	3,791/12,350	0.27	0.02	1,437/12,350	0.25	0.04	563/12,350	0.47	0.09

Supplementary Table 3: Heritability and genetic correlation estimates of BD I and BD II

Heritability and genetic correlation for BD I and BD II were compared in SWEBIC and BDRN as described in the text. The number of cases/controls listed in the N columns account for removal of individuals for cryptic relatedness > 0.05. The reported genetic correlations (and corresponding control sample sizes and standard errors) are the mean of the two middlemost values of 100 permutations, each permutation using the same set of cases and a randomly assigned half of the full control set(s) for the site(s) in the calculation (see Figure 3). Case/control counts for individual sites may differ from those in Supplementary Tables 1 and 2 due to cryptic relatedness filters being applied at different levels of resolution (i.e., across sites or within sites).

	**Subtype**	**Heritability**			**Genetic correlation**		
		N	SNP-h^2	SE	N	$r_{g\text{-}I/II}$	SE
SWEBIC	BD I	1,135/5,782	0.40	0.05	1,135/2,896	0.83	0.23
	BD II	607/5,782	0.23	0.08	607/2,886		
BDRN	BD I	1,678/5,384	0.37	0.04	1,678/2,691	0.88	0.18
	BD II	793/5,384	0.28	0.07	793/2,693		
SWEBIC-BDRN	BD I	2,811/11,164	0.35	0.02	2,813/5,582	0.77	0.10
	BD II	1,398/11,164	0.25	0.04	1,397/5,595		

Supplementary Table 4: Genome-wide significant loci in the ICCBD GWAS.

Summary statistics for the 2 loci identified as genome-wide significant in the ICCBD GWAS. The association values and odds ratios (OR) for these SNPs is also shown for the PGCBD study and the ICCBD-PGCBD meta-analysis.

Chr	SNP	Position	Allele	Genes in LD (R^2 >0.25)	ICCBD		PGCBD		ICCBD-PGCBD	
					p-value	OR	p-value	OR	p-value	OR
9	chr9:129209201:D	129,209,201	TC	*MVB12B, NRON, LMX1B*	2.48×10^{-8}	1.14	0.75	1.01	3.04×10^{-5}	1.07
10	rs10884920	111,774,807	A	*ADD3,LOC100505933,XPNPEP1*	1.20×10^{-8}	1.17	0.02	1.07	3.28×10^{-8}	1.12

Supplementary Table 5: Previously identified BD GWAS loci in ICCBD-PGCBD

Summary of ICCBD-PGCBD GWAS results for the 12 genome-wide significant loci previously reported in the 3 largest BD GWAS. Rows in gray were found to be genome-wide significant in ICCBD-PGCBD.

Locus	Previous Report(s)				ICCBD-PGCBD	
	Report(s)[a]	Chr	Region	Gene	SNP	P-value
1	1	1	79.2Mb	*PTGFR*	rs2211577	6.89×10^{-8}
2	1	2	97.5Mb	*LMAN2L*	rs56351161	3.19×10^{-10}
3	1,2	3	36.8Mb	*TRANK1*	rs9834970	1.59×10^{-10}
4	1	3	52.3Mb	Intergenic	rs2302417	2.75×10^{-9}
5	2	5	7.5Mb	*ADCY2*	chr5:7587236:D	2.25×10^{-4}
6	2	6	98.1Mb	*MIR2113, POU3F2*	rs76669370	N/A[d]
7	3	6	152.8Mb	*SYNE1*	rs7759578	4.95×10^{-8}
8	1,2,3	10	60-62Mb	*ANK3*	rs10994299	1.28×10^{-9}
9	2,3[b]	11	79Mb	*ODZ4*	rs73496688	1.86×10^{-7}
10	3[c]	12	2.4Mb	*CANCNA1C*	rs4765913	3.12×10^{-9}
11	3	12	47.8Mb	Many	rs7969091	2.33×10^{-8}
12	2	19	19.3Mb	*NCAN*	rs17751109	2.33×10^{-4}

[a]Numbers correspond to the following publications: *1* - Chen et al (6568 cases, 8187 controls; reference 26 in manuscript); *2* - Muhleison et al (9747 cases, 14278 controls; reference 27 in manuscript); *3* - PGCBD (7481 cases, 9250 controls; reference 24 in manuscript)

[b]Genome-wide significance was surpassed in both the primary PGCBD GWAS (7481 cases, 9250 controls) and in the combined meta-analysis of PGCBD and replication sample (11,974 cases, 51,792 controls)

[c]Genome-wide significance was surpassed only in the combined meta-analysis of PGCBD and replication sample (11,974 cases, 51,792 controls)

[d]SNP did not pass quality control filters for inclusion in the ICCBD-PGCBD meta-analysis

Supplementary Table 6: ICCBD-PGCBD loci by BD subtype.

Association results for the the 8 ICCBD-PGCBD genome-wide significant loci in BD subtypes in the ICCBD cohort. For each BD subtype analysis, the full ICCBD control sample (n=12,639) and the full case sample for the indicated subtype (ICCBD BD I n=3,888, ICCBD BD II n=1,457, ICCBD SAB n=591) were included to test for association. Results for these loci in the full ICCBD-PGCBD, PGCBD and ICCBD analyses (as presented in Figure 2) are also presented here for comparison, and the colors in the first 4 columns correspond to the colors in the table in Figure 2. For subtype association tests, standard errors (SE) are included due to the differences in case sample sizes.

				ICCBD-PGCBD		PGCBD		ICCBD		ICCBD BD I			ICCBD BD II			ICCBD SAB		
Chr	SNP	Position	Allele	p-value	OR	p-value	OR	p-value	OR	p-value	OR	SE	p-value	OR	SE	p-value	OR	SE
2	rs56361249	97380229	T	3.19×10^{-10}	1.12	4.71×10^{-7}	1.14	4.12×10^{-5}	1.10	9.82×10^{-4}	1.10	0.03	0.03	1.10	0.04	0.40	1.06	0.07
3	rs9834970	36856030	T	1.59×10^{-10}	0.90	5.75×10^{-6}	0.90	1.87×10^{-7}	0.89	1.46×10^{-6}	0.88	0.03	6.39×10^{-3}	0.90	0.04	0.04	0.87	0.07
3	rs2302417	52814256	T	2.75×10^{-9}	1.11	2.80×10^{-6}	1.12	4.46×10^{-5}	1.10	6.33×10^{-5}	1.12	0.03	0.03	1.09	0.04	0.22	1.09	0.07
6	rs1203233	152714606	G	4.46×10^{-8}	0.91	4.48×10^{-8}	0.88	1.83×10^{-3}	0.93	0.01	0.93	0.03	0.28	0.96	0.04	0.07	0.88	0.07
10	rs10994299	62076628	C	1.28×10^{-9}	0.81	2.42×10^{-10}	0.73	1.64×10^{-3}	0.86	3.54×10^{-4}	0.82	0.05	0.09	0.87	0.08	0.91	1.02	0.14
10	rs10884920	111774807	A	3.28×10^{-8}	1.12	0.02	1.07	1.20×10^{-8}	1.17	3.98×10^{-6}	1.16	0.03	2.66×10^{-3}	1.15	0.05	0.05	1.17	0.08
12	rs4765913	2419896	A	3.12×10^{-9}	1.13	4.40×10^{-6}	1.14	3.05×10^{-5}	1.12	1.70×10^{-4}	1.13	0.03	0.07	1.09	0.05	0.58	1.05	0.08
12	rs10459221	49466994	C	2.33×10^{-8}	0.91	6.98×10^{-8}	0.88	1.87×10^{-3}	0.93	4.09×10^{-4}	0.91	0.03	0.23	0.95	0.04	0.38	0.94	0.07

References

1 First MB, Spitzer RL, Gibbon M, Williams JBW. Structured Clinical Interview for DSM-IVo Axis I Disorders (SCID-I), Clinician Version, Administration Booklet. 2012.

2 Bergen SE, O'Dushlaine CT, Ripke S, Lee PH, Ruderfer DM, Akterin S *et al.* Genome-wide association study in a Swedish population yields support for greater CNV and MHC involvement in schizophrenia compared with bipolar disorder. *Mol Psychiatry* 2012; **17**: 880.

3 Green EK, Hamshere M, Forty L, Gordon-Smith K, Fraser C, Russell E *et al.* Replication of bipolar disorder susceptibility alleles and identification of two novel genome-wide significant associations in a new bipolar disorder case-control sample. *Mol Psychiatry* 2013; **18**: 1302–1307.

4 Pato MT, Sobell JL, Medeiros H, Abbott C, Sklar BM, Buckley PF *et al.* The genomic psychiatry cohort: partners in discovery. *Am J Med Genet B Neuropsychiatr Genet* 2013; **162B**: 306–12.

5 International Schizophrenia Consortium., Purcell SM, Wray NR, Stone JL, Visscher PM, O'Donovan MC *et al.* Common polygenic variation contributes to risk of schizophrenia and bipolar disorder. *Nature* 2009; **460**: 748.

6 Ripke S, O'Dushlaine C, Chambert K, Moran JL, Kähler AK, Akterin S *et al.* Genome-wide association analysis identifies 13 new risk loci for schizophrenia. *Nat Genet* 2013; **45**: 1150–9.

7 UK IBD Genetics Consortium, Barrett JC, Lee JC, Lees CW, Prescott NJ, Anderson CA *et al.* Genome-wide association study of ulcerative colitis identifies three new susceptibility loci, including the HNF4A region. *Nat Genet* 2009; **41**: 1330.

8 Schizophrenia Working Group of the Psychiatric Genomics Consortium. Biological insights from 108 schizophrenia-associated genetic loci. *Nature* 2014; **511**: 421–427.
